# Combined Analyses of Chloroplast DNA Haplotypes and Microsatellite Markers Reveal New Insights Into the Origin and Dissemination Route of Cultivated Pears Native to East Asia

**DOI:** 10.3389/fpls.2018.00591

**Published:** 2018-05-07

**Authors:** Xiaoyan Yue, Xiaoyan Zheng, Yu Zong, Shuang Jiang, Chunyun Hu, Peiyuan Yu, Guoqin Liu, Yufen Cao, Hongju Hu, Yuanwen Teng

**Affiliations:** ^1^Department of Horticulture, Zhejiang University, Hangzhou, China; ^2^The Key Laboratory of Horticultural Plant Growth, Development and Quality Improvement, Ministry of Agriculture of China, Hangzhou, China; ^3^Zhejiang Provincial Key Laboratory of Integrative Biology of Horticultural Plants, Hangzhou, China; ^4^College of Ecology, Lishui University, Lishui, China; ^5^College of Agriculture, Guizhou University, Guiyang, China; ^6^Research Institute of Pomology, Chinese Academy of Agricultural Sciences, Xingcheng, China; ^7^Institute of Fruit and Tea, Hubei Academy of Agricultural Sciences, Wuhan, China

**Keywords:** genetic variation, origin, dissemination route, microsatellite, chloroplast DNA, Asian pear

## Abstract

Asian pear plays an important role in the world pear industry, accounting for over 70% of world total production volume. Commercial Asian pear production relies on four major pear cultivar groups, Japanese pear (JP), Chinese white pear (CWP), Chinese sand pear (CSP), and Ussurian pear (UP), but their origins remain controversial. We estimated the genetic diversity levels and structures in a large sample of existing local cultivars to investigate the origins of Asian pears using twenty-five genome-covering nuclear microsatellite (simple sequence repeats, nSSR) markers and two non-coding chloroplast DNA (cpDNA) regions (*trn*L-*trn*F and *acc*D-*psa*I). High levels of genetic diversity were detected for both nSSRs (*H*_E_ = 0.744) and cpDNAs (*H*d = 0.792). The major variation was found within geographic populations of cultivated pear groups, demonstrating a close relationship among cultivar groups. CSPs showed a greater genetic diversity than CWPs and JPs, and lowest levels of genetic differentiation were detected among them. Phylogeographical analyses indicated that the CSP, CWP, and JP were derived from the same progenitor of *Pyrus pyrifolia* in China. A dissemination route of cultivated *P. pyrifolia* estimated by approximate Bayesian computation suggested that cultivated *P. pyrifolia* from the Middle Yangtze River Valley area contributed the major genetic resources to the cultivars, excluding those of southwestern China. Three major genetic groups of cultivated *Pyrus pyrifolia* were revealed using nSSRs and a Bayesian statistical inference: (a) JPs; (b) cultivars from South-Central China northward to northeastern China, covering the main pear production area in China; (c) cultivars from southwestern China to southeastern China, including Yunnan, Guizhou, Guangdong, Guangxi, and Fujian Provinces. This reflected the synergistic effects of ecogeographical factors and human selection during cultivar spread and improvement. The analyses indicated that UP cultivars might be originated from the interspecific hybridization of wild *Pyrus ussuriensis* with cultivated *Pyrus pyrifolia*. The combination of uniparental DNA sequences and nuclear markers give us a better understanding of origins and genetic relationships for Asian pear groups and will be beneficial for the future improvement of Asian pear cultivars.

## Introduction

Pear (*Pyrus* L.), as an important temperate fruit tree worldwide, has been cultivated for more than 3,000 years (Pu and Wang, 1963; Janick, 2000). *Pyrus* species are naturally distributed in the Eurasian continent and are geographically divided into two native groups: occidental and oriental pears (Bailey, 1917). The occidental pears, including over 20 species, occur in Europe, Northern Africa, Asia Minor, Iran, Central Asia, and Afghanistan, and the majority of cultivars in these areas originated from *Pyrus communis* L. (Rubtsov, 1944). The oriental pears, comprising 12–15 species, are distributed from the Tian Shan and Hindu Kush Mountains eastward to Japan and are largely concentrated in East Asia, including China, Japan, and Korea (Rubtsov, 1944; Challice and Westwood, 1973). The commercial pear cultivars native to East Asia can be mostly divided into four groups, Ussurian pear (UP; *P. ussuriensis* Maxim.), Chinese white pear (CWP; *Pyrus pyrifolia* White Pear Group, generally known as *P. x* *bretschneideri* Rehder), Chinese sand pear (CSP; *P. pyrifolia* Nakai), and Japanese pear (JP; *P. pyrifolia* Nakai), based on their geographic distributions, and morphological and physiological traits (Teng and Tanabe, 2004). In the limited areas of Gansu, Qinghai, and Xinjiang Provinces, Xinjiang pear (*P.* x *sinkiangensis* Yu) is cultivated, which is a hybrid of *P. communis* and *P. pyrifolia* (Teng et al., 2001). Asian pears account for over 70% of the worldwide total in terms of both production volume and cultivated area (FAO).

The four main cultivated pear groups native to East Asia, except JP, are native to China, and the total number of cultivars and landraces has been estimated as ∼3,000 in China (Pu and Wang, 1963). UP is the most cold-tolerant cultivar, mainly cultivated in northeast China, and is generally considered to be directly domesticated from the wild species *P. ussuriensis*. Its fruit are usually small globose or oblate with a persistent calyx, and they need an after-ripening process to become soft and edible like the European pear (Pu and Wang, 1963; Teng et al., 2002). CSP is distributed in southern China and was derived from wild *P. pyrifolia* in the Changjiang (Yangtze) River Valley of China. Its fruit, with smooth or russet skin, vary greatly in shape, size, and shelf life (Pu and Wang, 1963). CWP grows only between the areas that produce UP and CSP. Therefore, CWP was once proposed to have originated from the hybridization of *P. ussuriensis* and *P. pyrifolia* (Rubtsov, 1944; Kikuchi, 1946). In China, this group of cultivars has generally been assigned to *P*. x *bretschneideri* Rehder (Teng and Tanabe, 2004). The latter was named by Rehder (1915) based on seeds sent by Dr. Bretschneider from China. Some researchers speculated that *P*. x *bretschneideri* might have originated from the natural hybridization between *Pyrus betulifolia* Bunge and the large-fruited cultivars grown in North Hebei Province (Kikuchi, 1946; Challice and Westwood, 1973). Thus, *P*. x *bretschneideri* is different in the morphological characteristics from the cultivars of CWP (Kikuchi, 1946, 1948). Therefore, CWP cultivars should not have originated from *P*. x *bretschneideri* (Kikuchi, 1946, 1948). The cultivated pears native to Japan are referred to as JP and they have been considered to be from the same germplasm as *P. pyrifolia* (Teng and Tanabe, 2004). However, the origin of JP and its relationship with CSP are long standing controversies. Some Japanese horticulturists believe that JP cultivars were domesticated from wild *P. pyrifolia* that once grew in southern Japan (Kikuchi, 1948). However, there has been no solid evidence for the existence of wild *P. pyrifolia* in Japan (Kajiura and Suzuki, 1980). Others proposed that the progenitors of JP cultivars might have been introduced from China during ancient times (Shimura, 1988).

Over the last few decades, genetic analyses based on DNA markers, such as RAPD (Random Amplified Polymorphic DNA; Teng et al., 2001, 2002), AFLP (Amplified Fragment Length Polymorphism; Bao et al., 2008), and SSR (Simple Sequence Repeats; Bao et al., 2007; Iketani et al., 2012; Nishio et al., 2016) have advanced our understanding of the genetic diversity and genetic relationships among cultivated pears native to East Asia. CWP is closely related to CSP (Teng et al., 2002), and may be treated as an ecotype of *P*. *pyrifolia*: *P*. *pyrifolia* White Pear Group (Bao et al., 2007). Also, JP shares some identity with pears from China (Iketani et al., 2012), especially cultivars from Zhejiang Province (ZJ) and Fujian Province (FJ) (Teng et al., 2002; Bao et al., 2007; Song et al., 2014), which was supported by our recent studies based on retrotransposons markers (Jiang et al., 2015, 2016). Thus, the three major pear cultivar groups (CSP, CWP, and JP) native to East Asia appear to have evolved from the same germplasm: wild *P*. *pyrifolia* in China (Jiang et al., 2015, 2016). Unfortunately, no population of wild *P. pyrifolia* still exists. Therefore, we cannot elucidate the origins and levels of geographic differentiation of CSP, CWP, and JP by comparing their genetic makeup with those of wild progenitors, as achieved with apple (Cornille et al., 2012; Nikiforova et al., 2013) and grape (Myles et al., 2011). In addition, most of the aforementioned studies were based on either relatively small or geographically limited samples that under-represented the rich diversity of cultivated pears native to East Asia. There are still some questions that need to be addressed: Where were the *P. pyrifolia* initially cultivated? How did pear cultivars genetically diverge during the spread of the germplasm? Are there varied genetic differentiation levels among different cultivar groups, especially the three cultivated *P. pyrifolia* groups? Thus, additional studies are required to answer these questions to better understand the origins and dissemination route of cultivated Asian pears. A better understanding of genetic diversity of existing local cultivars will be helpful for developing new cultivars with the combination of good fruit quality and resistance to abiotic and biotic stresses, and providing information for efficient conservation of pear germplasm resources.

In this study, 441 pear accessions from different geographical regions were collected across China and Japan, covering the major distribution areas of pear cultivars or landraces. Two hypervariable non-coding chloroplast DNA regions, *trn*L-*trn*F and *acc*D-*psa*I, which have been widely applied in molecular phylogeny and genetic diversity studies of the genus *Pyrus* (Kimura et al., 2003; Liu et al., 2013; Wuyun et al., 2013; Zheng et al., 2014; Zong et al., 2014), were adopted to elucidate the evolutionary history of pear cultivars using a phylogeographic approach. Meanwhile, markers of genome-covering nuclear loci containing simple sequence repeats (nSSRs; microsatellites) were employed to investigate the genetic structure of East Asian cultivated pears using a Bayesian statistical inference based on the theory of population genetics that used no prior information on sample origins. This has been widely used for analyzing genetic structures of fruit crops, such as pear (Iketani et al., 2012), apple (Robinson et al., 2001; Cornille et al., 2012), and grape (Bacilieri et al., 2013). The combination of data from maternally inherited genetic markers (cpDNA sequences) and parentally inherited nuclear markers (nSSRs), will help us achieving a comprehensive understanding of genetic diversity of Asian pears and provide important clues on the origin and dissemination route of Asian pear cultivars.

## Materials and Methods

### Plant Materials and DNA Extractions

A total of 441 pear accessions collected from different geographic areas in East Asia were analyzed in this study (Supplementary Table S1). All cultivated *P. pyrifolia* accessions (*n* = 401), including 221 CSPs and 125 CWPs, and 55 JPs were assigned to 14 geographic populations based on their geographical origins, of which CSPs included seven geographical populations of cultivated pears from south of Yangtze river in China, CWPs included five geographical populations from north of Yangtze river in China, and JPs contained two geographical populations from Japan, and the population code of geographical populations were described in Supplementary Table S1. Forty *P. ussuriensis* accessions, including 22 cultivated *P. ussuriensis* (UPs) and 18 wild *P. ussuriensis* (WUPs) sampled from North and Northeast China, were also used for the genetic analysis. Most of the local cultivars were sampled from different pear germplasm repositories in China and Japan, and a few were collected *in situ* (Supplementary Table S1). DNAs of all pear accessions were extracted from young leaves using a modified cetyl trimethyl ammonium bromide protocol (Doyle, 1987).

### Nuclear Microsatellite Amplification

The pear accessions were genotyped using 25 polymorphic nSSR loci distributed across the genome (Chen et al., 2014; Supplementary Table S2). The 25 nSSR loci were selected based on the degree of applicability and number of polymorphisms in different *Pyrus* species. PCR amplification of microsatellite primers, including BGT23b, KU10, KA14, 28f4, 02b1 and “MES”, were performed as described in previous protocols (Guilford et al., 1997; Yamamoto et al., 2002a,b; Yao et al., 2010), with fluorescent dye-labeled forward primers. The other recently developed nSSR primers (“NAU,” “CTG,” and “TXY”) were used in an economic method for the fluorescent labeling of PCR products (Schuelke, 2000), and the amplification programs were performed using protocols previously described by Yue et al. (2014). Genotyping was performed on an ABI 3700XL Genetic Analyzer (Applied Biosystems, United States), with an internal size standard (GeneScan^TM^ 500 LIZ, Applied Biosystems). Alleles were scored using GENEMAPPER 4.0 software (Applied Biosystems).

### Genetic Analysis of Microsatellites

Genotyping errors and null alleles were confirmed by MICROCHECKER (Van Oosterhout et al., 2004). The number of alleles (*N*), number of observed alleles (*Na*), number of effective alleles (*Ne*), Shannon’s Information Index (*I*), expected heterozygosity (*H*_E_), and observed heterozygosity (*H*_O_) were calculated for each nSSR locus and geographic populations by GENEPOP4.0 (Raymond and Rousset, 1995). The number of unique alleles was estimated by GenAIEx 6.5 (Peakall and Smouse, 2012). Analyses of molecular variance (AMOVA) were conducted by Arlequin 3.5 (Excoffier and Lischer, 2010) with standard AMOVA computations and 2,000 permutations using nSSR data to assess the levels of genetic differentiation among cultivar groups and among populations with different groupings. Allele frequency-based pairwise *F*_ST_ index with 1,000 permutations was calculated by use of Arlequin 3.5 (Excoffier and Lischer, 2010), to describe the nuclear genetic differentiation among different populations. The software STRUCTURE2.3.3 (Pritchard et al., 2000) was applied to the multilocus nSSR data of East Asian pear accessions to infer the different gene pools (*K*) in the data set. Genotypic clustering was implemented in STRUCTURE using the admixture model with independent allele frequencies without using sites or populations of individuals as priors as described by Richards et al. (2009), and 10 independent runs were performed for each *K* in the range of 2–16 with 200,000 MCMC (Markov Chain Monte Carlo) iterations after a burn-in of 200,000 steps. The results were uploaded to the STRUCTURE HARVESTER web site (Earl, 2012) to estimate the optimal value of *K* based on Evanno et al.’s (2005) criterion of Δ*K*, which used the rate of change in the log probability of data between successive *K*-values. In addition, CLUMPP (Jakobsson and Rosenberg, 2007) software was employed to average the 10 independent simulations and DISTRUCT (Rosenberg, 2004) was applied to illustrate the results graphically. To further clarify the genetic diversity of pear accessions in each geographic population, pear genotypes were assigned to a certain group when 85% or more of their inferred genome belonged to that group. The others, with lower scores, were considered admixed genotypes as described by Bacilieri et al. (2013).

### Amplification and Alignment of Chloroplast Non-coding DNA Regions

The *trn*L-*trn*F and *acc*D-*psa*I intergenic regions were amplified and sequenced for all pear accessions listed in Supplementary Table S1. Amplification and sequencing conditions of the two cpDNA fragments were as described in a previous study (Zheng et al., 2014). Two cpDNA regions were combined for haplotype identification and phylogeographic analysis. The haplotypes of cpDNA fragments were identified and named following a previous phylogenetic study in *Pyrus* (Zheng et al., 2014). Chloroplast DNA sequences were aligned by ClustalX 1.81 (Thompson et al., 1997) and refined manually. Each indel (insertion or deletion) was treated as a single mutation event, regardless of its size, and coded as a substitution (A or T) owing to their equal probability levels (Chen et al., 2008).

### Genetic Analyses of Chloroplast DNA

Haplotype number (*H*), haplotype diversity (*H*d), number of segregating sites, nucleotide diversity, and the average number of nucleotide differences (*K*) were calculated by DnaSP 5 (Librado and Rozas, 2009). AMOVA were conducted to assess the levels of genetic differentiation among and within different groupings using 20,000 permutations of standard AMOVA computations by Arlequin3.5 (Excoffier and Lischer, 2010). Chloroplast DNA genetic differentiation was examined for geographical populations by a classical analysis of variance calculating haplotype frequency-based *F*_ST_, and the existence of a significant genetic differentiation among populations was determined by permutation analyses using 1000 randomly permuted data sets with Arlequin3.5 (Excoffier and Lischer, 2010). A Bayesian clustering of cpDNA data was conducted by BAPS6.0 (Corander et al., 2007). The maximum number of populations was set as 10 replicates for 2–16 during mixture analyses. The other parameters for admixture analyses based on the mixture analyses were set as default.

### Phylogeographic Analyses of cpDNA Haplotypes

Two differentiation indices (*G*_ST_ and *N*_ST_) were computed for haplotypes in geographic populations and compared by a test with 1,000 permutations using the program Permut 2.0 (Pons and Petit, 1996). *G*_ST_ is measured by simply making use of the allelic frequency to explain the genetic variation among all populations. *N*_ST_ is considered to represent the similarity levels between the haplotypes, explaining the genetic differentiation influenced by both haplotype frequencies and genetic distances between haplotypes (Pons and Petit, 1996). There would be a phylogeographic structure among geographic populations, if the value of *N*_ST_ is significantly greater than the value of *G*_ST_ (Pons and Petit, 1996). Haplotype networks were constructed for the combined cpDNA haplotypes of cultivated pears using the computer program TCS 1.21 (Clement et al., 2000) with the 95% statistical parsimony criterion. The frequency of each haplotype in the TCS network was used to estimate haplotype outgroup probabilities, which correlated with the haplotype age (Clement et al., 2000). Neighbor-Net splits graphs (NN graphs) of cpDNA haplotypes were constructed using SplitsTree (Huson and Bryant, 2006) with the criterion set to the uncorrected *p* distance. Haplotype sequences of wild species *Pyrus pashia* 3 (tH4aH12), *P. pashia* 7 (tH5aH14), and *P. betulifolia* 1 (tH3aH7), as well as haplotypes of outgroup *Malus domestica* ‘Ralls’ (tH19aH34) and *M. rockii* (tH17aH33) (Zheng et al., 2014) were included in the NN graph analysis.

### Estimation of the Dissemination Route of Cultivated *P. pyrifolia*

To further explore the dissemination route of cultivated *P. pyrifolia*, pear accessions from 14 geographic populations were assigned into 10 Pops, according to their geographic origins and genetic similarities. The dissemination route of cultivated *P. pyrifolia* was estimated from cpDNA haplotypes by approximate Bayesian computation using the software DIYABC (Cornuet et al., 2014). Two estimates, including a direct estimate and a logistic regression estimate, were computed to determine the more supported scenario of the evolutionary route of cultivated *P. pyrifolia* in East Asia. The former estimate simply represents the number of times that a certain scenario was detected in the data sets that were simulated from the historical models. The latter was a polychotomic weighted logistic regression, which was performed on the simulate data sets with dependent variables from a proportion of certain scenarios in the simulated data sets and independent variables from the differences of summary statistics between observed and simulated data sets (Cornuet et al., 2014).

## Results

### Diversity of Asian Pears Inferred From nSSR Loci

Twenty-five nSSR loci displayed a high level of polymorphism in Asian pears with an average of 24 alleles per locus and ranging from 13 to 43 alleles (Supplementary Table S2). Genome-derived nSSRs generally displayed higher polymorphism levels than EST-derived or transcriptome-derived nSSR markers. The *H*_E_ and *H*_O_ values of geographical cultivar populations ranged from 0.660 (JPa) and 0.551 (JPb), respectively, to 0.792 (UP) and 0.665 [Sichuan Province (SC)], respectively, and the overall means of East Asian pears were 0.744 and 0.603, respectively (**Table 1**). Geographic populations of Fujian Province (FJ; 10) and Yunnan Province (YN; 11) displayed the largest numbers of unique alleles. The inbreeding coefficient ranged from 0.083 to 0.285, which was close to zero and consistent with the self-incompatibility system of pears.

**Table 1:** Summary of genetic variation in East Asian pears based on nuclear microsatellite loci.

Group code	Population code	Number of cultivars	*Na*	*Ne*	*I*	*H*_O_	*H*_E_	Unique alleles	*F*_IS_
**Cultivated *P. pyrifolia***		
JP	JPa	29	6.96	3.583	1.394	0.604	0.66	4	0.083
	JPb	26	8.92	4.121	1.629	0.551	0.718	7	0.236
CSP	ZJ	27	10.36	5.559	1.876	0.567	0.782	5	0.285
	FJ	34	12.08	5.746	1.956	0.612	0.79	10	0.234
	GG	35	10.76	5.512	1.885	0.582	0.781	2	0.264
	GZ	32	9.56	4.643	1.719	0.598	0.744	7	0.207
	YN	30	10.16	5.256	1.791	0.627	0.759	11	0.177
	SC	29	10.84	5.014	1.857	0.665	0.774	7	0.148
	HHJ	34	10.36	4.929	1.783	0.629	0.753	7	0.178
CWP	AJ	26	9.24	4.674	1.707	0.609	0.735	2	0.177
	GQ	29	10.4	4.778	1.779	0.623	0.743	8	0.184
	SS	27	8.72	4.617	1.655	0.613	0.725	4	0.181
	SHH	32	9.84	5.02	1.735	0.585	0.731	3	0.201
	JL	11	6.08	3.96	1.47	0.613	0.697	1	0.112
**Cultivated *P. ussuriensis***		
UP	UP	22	10.52	6.218	1.94	0.62	0.792	10	0.234
**Wild *P. ussuriensis***		
WUP	WUP	18	8.6	4.634	1.66	0.55	0.714	16	0.253
Mean			9.59	4.891	1.74	0.603	0.744	6.5	0.197

*Population codes are identified in Supplementary Table S1. Na, observed mean number of alleles; Ne, effective number of alleles; I, Shannon’s Information index; H_E_, expected heterozygosity; H_*O*_, observed heterozygosity. Unique alleles are displayed as absolute count values in the geographic populations. F_*IS*_, inbreeding coefficient. JP, Japanese pear; CWP, Chinese white pear; CSP, Chinese sand pear; UP, Ussurian pear*.

Analysis of molecular variance based on nSSR data was analyzed for pear cultivars and wild *P. ussuriensis* accessions native to East Asia (Supplementary Table S3). For different groupings of pear accessions, most of the total variation (88.91–93.18%) was partitioned within geographic populations. The highest variation (10.31%) among populations was observed among *P. ussuriensis* accessions (UP and WUP). Comparatively, the least of total variation (4.32%) was identified among populations within cultivar groups. Based on the *F*_ST_ index value (Wright, 1978), levels of nuclear genetic differentiation were generally low for comparisons among adjacent geographical populations of cultivated pears (0.015–0.0621), but fairly high between populations of cultivar groups and the group of wild *P. ussuriensis* accessions (0.10306–0.18228) (Supplementary Table S9).

### Genetic Structure of Asian Pears

**FIGURE 1:**
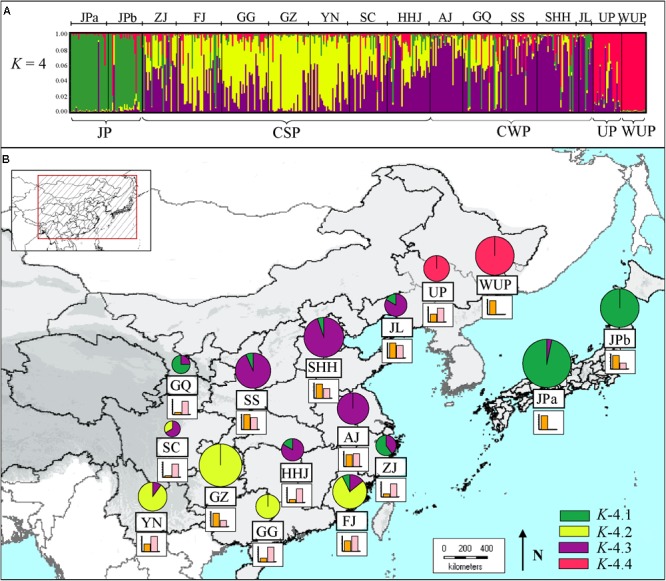
**(A)** Estimated genetic structures for Asian pears based on microsatellite data using a Bayesian modeling approach. The figure is shown for *K* = 4, which represents the estimated gene pools of pear accessions. Each individual is represented by a vertical colored line. The short black lines on the top of the figure indicate the separation of pears based on different geographic origins, which are labeled on the top of the figure. Population codes were identified in Supplementary Table S1. **(B)** Genetic compositions of geographic populations for *K* = 4 by Bayesian modeling approach based on microsatellite data. The histograms under the pie charts represent the proportion of “non-admixed” (orange) versus “admixed” genotypes (pink) in a certain geographic population. For “non-admixed” genotypes, the pie slices represent the proportion of gene pools in each geographic population. The colors correspond to those of the gene pools identified in **(A)**. The size of the pie charts approximately correspond to the number of non-admixed genotypes in geographic population.

STRUCTURE results for the nSSR dataset (*n* = 441), based on the likelihood output of the genetic admixture analysis and Evanno’s Δ*K* statistics indicated that *K* = 4 was the most pertinent levels of population subdivision (Supplementary Figure S1). At *K* = 4 (**Figure 1A**), two JP populations were dominated by a gene pool, whereas CSP populations were mainly comprised of two gene pools, and CWP populations were also dominated by two gene pools. The cultivated UPs were composed of one main gene pool, and two minor gene pools, whereas WUP accessions were predominantly comprised of a single gene pool.

Using a threshold of >85% for genetic group assignation, a total of 195 non-admixed genotypes (out of 441 pear accessions) were assigned to four genetic groups (Supplementary Table S4). The other 246 cultivars were indicated as the admixed genotypes, which accounted for 58.05% of the total number of pear accessions. As seen in **Figure 1B**, the proportion of non-admixed genotypes displayed great differences among the geographic-based populations and was generally higher in northern populations [i.e., Shandong, Henan, and Hebei Provinces (SHH), and Jilin and Liaoning Provinces (JL)], and JP populations [western Japan (JPa) and northern Japan (JPb)]. Comparatively, admixed genotypes accounted for the largest proportions of geographic-based cultivar populations of southwestern China, northwestern China and the southernmost China, such as SC (89.66%), GQ (89.66%), and Guangdong and Guangxi Province (GG; 82.86%). The proportion of admixed genotypes generally decreased as the distance increased from these western and mid-southern regions, especially in the JP population that had a very low admixed genotype level (3.45%), which was generally in accordance with the lower genetic diversity detected in these areas (**Table 1**).

The identified four genetic groups of Asian pear accessions displayed a distinct geographic pattern for their genetic structures (**Figure 1B**). The first group, *K*-4.1 (Supplementary Figure S2), contained almost all JP genotypes (45 of 55 cultivars), including 27 cultivars of JPa and 18 of JPb, as well as three cultivars from Zhejiang province (ZJ) and GQ, and one from FJ. The second group, *K*-4.2, was mainly composed of genotypes from the Guizhou province (GZ; 22), and southernmost population in GG (7) and FJ (11). The third group, *K*-4.3, included pears belonging to CWP and CSP mainly from mid-southern, eastern and northern populations, such as HHJ, Anhui and Jiangsu Provinces (AJ), Shaanxi and Shanxi Provinces (SS), SHH, and JL. The fourth group, *K*-4.4 included 36.36% of the UP cultivars and all the WUP accessions.

### Classification and Diversity of cpDNA Haplotypes

Sequence lengths of the combined alignments of *trn*L-*trn*F and *acc*D-*psa*I ranged from 1,486 to 1,778 base pairs. In total, 10 nucleotide substitutions (5 in *trn*L-*trn*F and 5 in *acc*D-*psa*I; Supplementary Table S6) and 8 insertion and deletion (indels; 2 in *trn*L-*trn*F and 6 in *acc*D-*psa*I) were identified in combined cpDNA alignments, leading to 23 unique haplotypes (H1–H23) in 441 East Asian pears (Supplementary Table S1), including 7 tH haplotypes detected by sequences of *trn*L-*trn*F and 11 aH haplotypes discovered by alignments of *acc*D-*psa*I. A high level of *H*d (0.792) was detected in total pear accessions (**Table 2**). The number of segregating sites ranged from 6 to 11. The nucleotide diversity ranged from 1.2 (FJ) to 1.76 (SS) and the *K*-value among whole sequences is 3.097, ranging from 1.634 (WUP) to 3.122 (SS).

**Table 2:** Statistical summary of chloroplast DNA haplotype diversity in East Asian pears.

Group code	Population code	Number of cultivars	*H*	*H*d	*S*	π (×10^-3^)	*K*
**Cultivated *P. pyrifolia***		
JP	JPa	29	5	0.663	7	1.36	2.423
	JPb	26	3	0.698	7	1.27	2.255
CSP	ZJ	27	5	0.681	6	1.31	2.336
	FJ	34	6	0.771	6	1.2	2.138
	GG	35	7	0.734	7	1.44	2.556
	GZ	32	6	0.523	6	1.33	2.357
	YN	30	8	0.722	8	1.59	2.818
	SC	29	8	0.778	7	1.66	2.946
	HHJ	34	5	0.574	9	1.59	2.827
CWP	AJ	26	5	0.576	7	1.56	2.767
	GQ	29	5	0.515	10	1.24	2.202
	SS	27	6	0.809	9	1.76	3.122
	SHH	32	6	0.719	9	1.87	3.327
	JL	11	2	0.509	6	1.72	3.055
**Cultivated *P. ussuriensis***		
UP	UP	22	5	0.613	10	1.7	3.028
**Wild *P. ussuriensis***		
WUP	WUP	18	6	0.562	11	1.7	1.634
Total		441	24	0.792	14	1.74	3.097

*H, haplotype number; Hd, haplotype diversity; S, number of segregating sites; π, nucleotide diversity; K, average number of nucleotide differences between whole sequences. JP, Japanese pear; CWP, Chinese white pear; CSP, Chinese sand pear; UP, Ussurian pear.*

Of these 23 haplotypes, 19 haplotypes were detected in cultivated pears and four haplotypes (H20, H21, H22, and H23) only in the WUP accessions (Supplementary Table S7). In total, 16 haplotypes were presented in CSP, 9 in CWP, 6 in WUP, 5 in JP and 5 in UP. Seven (H4, H5, H8, H11, H15, H17, and 18) were discovered only in CSP, and H19 and H1 were discovered only in CWP and UP, respectively. Comparatively, no unique haplotype was detected in JP. Among the 19 cpDNA haplotypes from cultivated pears, haplotypes H3, H6, H7, and H14 had global frequencies in cultivated pears, covering all four Asian cultivar groups and accounting for 96.36% in JP, 91.2% in CWP, 82.8% in CSP, and 81.73% in UP of the total haplotypes, respectively (Supplementary Table S8). CSP and CWP shared seven haplotypes H3, H6, H7, H10, H12, H13, and H14; UP shared the common haplotypes H14 and H6 with CSP, CWP, and JP. Contrary to the cultivars of UP, which were domesticated directly from WUP, the most common haplotype H20 (tH1aH6) in WUP has not been detected in any cultivated UP. Cultivated UPs only shared the haplotype H1 with WUP accessions. Additionally, the rare haplotype H2 was shared by CWP and JP, but absent in CSP; H9 was shared by CSP and JP, but absent in CWP; H16 was shared by CSP and UP, but absent in JP and CWP.

Analyses of molecular variance analyses based on cpDNA data indicated there was 42.61–84.31% of total genetic variation partitioned within geographic populations (Supplementary Table S3). Higher levels of variation were observed among species (19.18%) or among populations (57.39%) in the groupings including wild *P. ussuriensis* accessions (WUP) than that were identified among cultivar groups [Cultivated *P. pyrifolia* and cultivated *P. ussuriensis*, 7.81%; cultivar groups of *P. pyrifolia* (JP, CSP and CWP), 3.64%]. The value of *F*_ST_ index for comparisons between populations of cultivated pears and WUP were generally high (0.554 to 0.657; Supplementary Table S9). They were fairly high (0.1251 to 0.4319) between geographical populations from southwestern (i.e., YN, SC, and GZ) and populations from other regions, including mid-southern (i.e., HHJ), eastern (i.e., ZJ) and northern (i.e., JL), as well as from Japan (JPa and JPb). In contrast, the values were small (-0.065 to 0.089) for comparisons between populations from southwestern or between populations from mid-southern, eastern, northern and Japan.

### Bayesian Clustering of Chloroplast DNA Data

**FIGURE 2:**
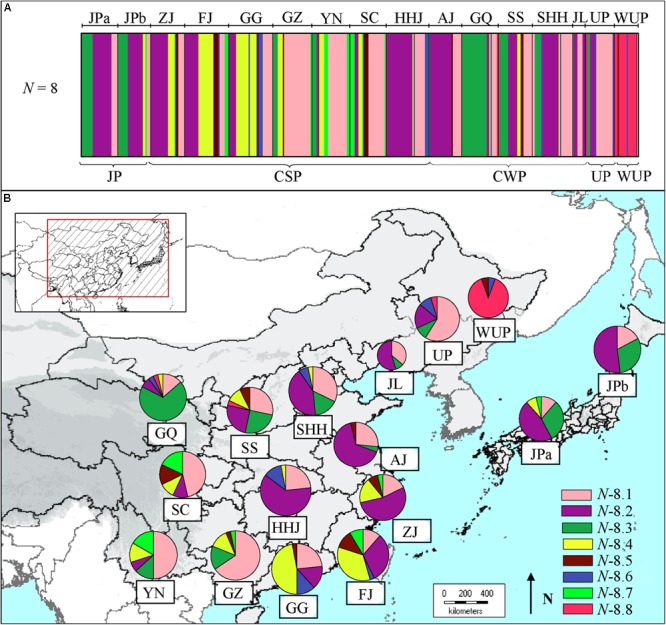
**(A)** Estimated genetic structures for Asian pears based on chloroplast DNA data using a Bayesian clustering and **(B)** genetic compositions of geographic populations. The size of the pie charts approximately corresponds to the number of genotypes in geographic population. The eight clusters are represented by different colors, as identified in Supplementary Table S5.

The Bayesian clustering based on cpDNA data revealed eight clusters (admixed populations) in East Asian pear accessions (**Figure 2A**), of which cluster *N*-8.8 contained 16 wild *P. ussuriensis* accessions and a few cultivars from north populations. Comparatively, the first four clusters (*N*-8.1 to *N*-8.4) included most of the cultivars (Supplementary Table S5). Cluster *N*-8.1 contained pear cultivars (*n* = 128) covering all geographical populations (**Figure 2B**), especially those from populations GZ (21), YN (15), SC (13) and UP (13). Cluster *N*-8.2 (*n* = 133) were mainly comprised of cultivars from mid-southern, eastern and northern populations [i.e., HHJ (21), AJ (17), ZJ (15), SHH (13) and JL (6)], as well as Japanese populations JPa (15) and JPb (12). Cluster *N*-8.3 contained pear cultivars (*n* = 62) mainly from population GQ (20), and a few from populations SS (7), JPa (9) and JPb (8). Genotypes in cluster *N*-8.4 were mainly comprised of cultivars (*n* = 52) mainly from southernmost populations GG (12) and FJ (16). Comparatively, cluster *N*-8.7 mainly included cultivars (*n* = 16) from populations of south China [i.e., YN (5), SC (5) and FJ (3)], while cultivars in cluster *N*-8.5 (*n* = 15) and *N*-8.6 (*n* = 16) were scattered in different geographic populations from south or north China.

### Phylogeography of cpDNA Haplotypes

**FIGURE 3:**
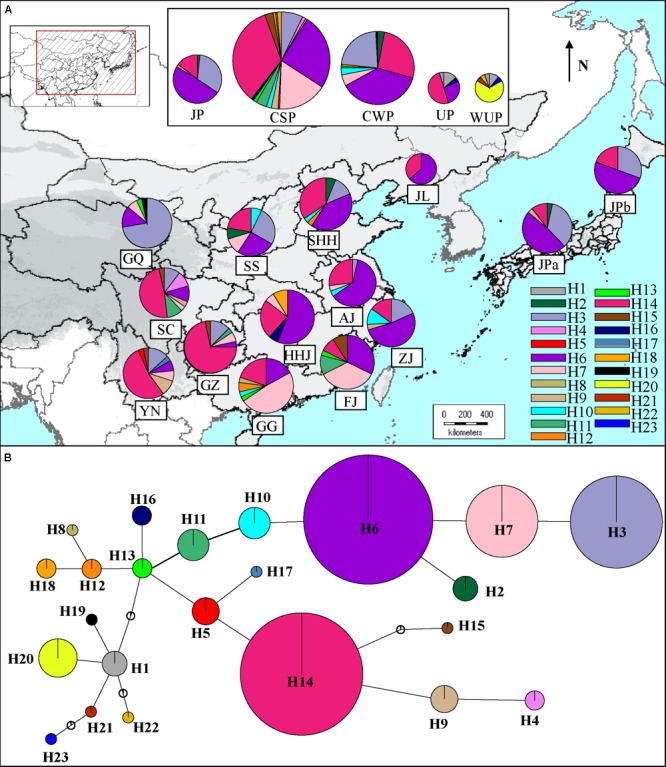
**(A)** Distribution of 23 haplotypes in geographic populations of cultivated *Pyrus pyrifolia* and Ussurian pears (UP and WUP). The 23 haplotypes, H1–23 are represented by different colors. The pie slices represent the proportion of different haplotypes in geographic population. The size of each circle approximately corresponds to the sample size (*n*) of the geographic population. **(B)** TCS-derived network based on combined cpDNA haplotypes. The circles represent different haplotypes. H1–23 represent haplotypes detected in Asian pear accessions. The small open circles represent missing haplotypes. The size of each circle is approximately proportional to the size of samples (*n*) harboring a certain haplotype, with the smallest circles representing *n* = 1 and the largest representing *n* = 134.

The number of haplotypes in geographic populations ranged from 3 (JPb) to 8 (YN and SC) (Supplementary Table S7). The two differentiation indexes showed lower values (*N*_ST_ = 0.202; *G*_ST_ = 0.162). A permutation test demonstrated that *N*_ST_ was significantly greater than *G*_ST_, indicating the existence of a phylogeographic structure in East Asian pear geographic populations (*P* < 0.01). To estimate the phylogeographic history of cultivated Asian pears, the TCS-derived network of haplotypes detected in all of the cultivated pears was constructed (**Figure 3B**). The results showed that two of the most frequent haplotypes (H14 and H6) corresponded to two major haplotype lineages in the TCA network and that haplotype H13 occupied a central position. The common haplotypes H7 and H3 were closely associated to haplotype H6, and haplotype H14 was surrounded by the rare haplotypes H4, H5, H9, H15, and H17. As shown in **Figure 3A**, haplotype H14 was very prevalent in southwestern populations (i.e., SC, YN, and GZ), and its frequency was decreased in southern, eastern and northern areas, while H6 was the most frequent haplotype in mid-southern (i.e., HHJ), eastern (i.e., ZJ) and northern populations (i.e., JL), as well as JP populations (i.e., JPa and JPb). Haplotypes H4, H5, H9, H15, and H17 located near H14 in the TCS network were largely confined to southern populations; haplotypes H3, H7, H10, and H11 (located in the same haplotype lineage of H6 in the TCA network) coexisted with H6, occurring across the eastern and northern areas (**Figure 3A**). Among these haplotype H3 was largely detected in the northwestern population GQ, and haplotype H7 was primarily detected in southern populations (i.e., FJ and GG). The central haplotype H13 was only detected in a few cultivars from populations GQ, FJ, and GG. Haplotypes H1 and H19 were only detected in northern geographic populations (i.e., GQ) and cultivated UPs.

A Neighbor-Net splits graph of haplotypes was constructed for East Asian cultivated pears and related wild species (Supplementary Figure S3). Many parallel edges were observed in the splits of the NN graph, indicating homoplasy among the combined haplotypes. Haplotype H13, in the inner network, was linked to the outside group (cpDNA haplotypes of *acc*D-*psa*I and *trn*L-*trn*F identified in *Malus* species), and most of the haplotypes were detected in pear accessions, which was consistent with the TCS network (**Figure 3B**). Haplotypes H1 and H19 were linked with four unique haplotypes of WUP, forming a separate lineage, and haplotypes H4, H5, H9, H14, H15, and H17 displayed the closest relationships to the two unique haplotypes of *P. pashia*.

### Putative Dissemination Route of Cultivated *P. pyrifolia* Inferred From cpDNA Data

Populations SC and YN, GQ, GZ, HHJ, AJ, ZJ, JPa and JPb, FJ and GG, SS, and SHH and JL were treated as Pop 1–10, respectively. Populations SC and YN, populations GG and FJ, populations SHH and JL, as well as populations JPa and JPb, were combined separately, since they were not only geographically adjacent and dominated by the same cpDNA haplotype and had the similar composition of gene pool inferred from STRUCTURE analysis based on nSSR data. Three putative historical models of Asian cultivated *P. pyrifolia* were constructed according to cpDNA haplotype variations among the geographic populations (**Figure 2B**). As a result, the estimates indicated that the first scenario was more likely to be the evolutionary route of cultivated *P. pyrifolia* in East Asia (Supplementary Figure S4). Under this scenario (Supplementary Figure S5), Pop 1 (SC and YN) and Pop 4 (HHJ) with constant effective population size N1 and N4, respectively, diverged at time t9 from an ancestral population of size NA, and Pop 3 (GZ) diverged from Pop 1 at time t8. Then, Pop 5 (AJ) and Pop 6 (ZJ) diverged from Pop 4 at time t7 and t6, respectively. Pop 7 (JP) diverged from Pop 6 (ZJ) at time t5, and Pop 8 (FJ and GG) and Pop 9 (SS) independently diverged from Pop 4 at times t4 and t3, respectively. Finally, Pop 2 (GQ) diverged from Pop 9 (SS) at time t2, and Pop 10 (SHH and JL) diverged from Pop 5 at time t1.

## Discussion

### High Levels of Genetic Diversity in Asian Pear Cultivars Across the Whole of China and Japan

In this study, more than 400 local pear cultivars were collected across the main pear distribution areas of China and Japan (Supplementary Table S1), and used in the genetic analysis. The sample size was much larger than any previous related studies (e.g., Bao et al., 2007, 2008; Yao et al., 2010; Cao et al., 2012; Iketani et al., 2012; Song et al., 2014; Jiang et al., 2015; Nishio et al., 2016). Twenty-five genome-covered nSSR loci in this study revealed a high level of genetic diversity (*H*_E_ = 0.744) in Asian pears, which was similar to that (0.73) reported by Liu et al. (2015), and much higher than that (0.64) reported by Song et al. (2014). Genetic analyses of geographic populations revealed that the levels of genetic diversity in southern populations (south of the Yangtze River) were generally greater than those in northern populations (north of the Yangtze River) (**Table 1**). Therefore, the conservation priority should be given to local cultivars from southern areas. The cultivars from geographic populations with high levels of genetic diversity are also useful genetic resources for future pear breeding programs (Kumar et al., 2017). A decrease in genetic diversity was also identified in two geographic populations in Japan, in agreement with previous studies (Bao et al., 2007; Iketani et al., 2012). In the future, cultivars with different genetic background, especially from South China should be applied in Japanese pear breeding programs to broaden the genetic diversity of Japanese pears as suggested by Nishio et al. (2016). Additionally, two cpDNA intergenic fragments displayed high degrees of genetic diversity (*H*d = 0.792) among Asian pear accessions, which was similar to that in the wild pear species *P. betulifolia* (*H*d = 0.807) (Zong et al., 2014) and higher than that in wild *P. pashia* (*H*d = 0.718) (Liu et al., 2013). The generally high levels of genetic diversity observed in Asian pear cultivars may be attributed to the large number of local cultivars and effective nSSR markers used in our studies compared with previous studies.

Wild *P. pyrifolia* species with large fruit, having five carpels and russet, yellow, or smooth green skin, were supposed to have originated in the Yangtze River Valley and the adjoining southern area (Pu and Wang, 1963). However, no populations of wild *P. pyrifolia* still exist in either China or Japan, possibly resulting from habitat destruction and land overexploitation (Heywood et al., 2007). Therefore, we cannot investigate the origins of cultivated *P. pyrifolia* by comparing *P. pyrifolia* cultivars with their wild ancestors. Instead, we collected as many local pear cultivars across the major areas of China and Japan as possible to cover all genetic variations. In addition, most haplotypes of wild oriental *Pyrus* species identified by Zheng et al. (2014) were detected in the cultivated pears, including all seven tH (haplotypes of *trn*L-*trn*F) and 11 aH (haplotypes of *acc*D-*psa*I), as well as two newly identified tH haplotypes [tH20 (MH010656) and tH21(MH010657)] and three newly identified aH haplotypes [one in cultivated Asian pears firstly reported in this study [aH36 (MG751777)] and two in WUP accessions [aH37 (MH010658) and aH38 (MH010659)]. The high levels of genetic diversity in cultivated *P. pyrifolia* based on nSSRs indicated that the enriched level of genetic diversification of cultivated Asian pears was represented by the Asian pear cultivars used in this study.

### Poor Differentiation Among Cultivated Asian Pear Groups

Cultivated pears worldwide are traditionally divided into two large geographic types, soft-fleshed European pears (*P. communis* L.) and the crisp-fleshed Asian pears (Janick, 2000), with a high-level genetic differentiation between them (Kumar et al., 2017). Asian pears contains two or three species that are generally classified into four major cultivar groups, including UP (*P. ussuriensis* Maxim.), CWP (*P. pyrifolia* CWP group, also traditionally assigning to *P.* x *bretschneideri*), CSP (*P. pyrifolia* Nakai), and JP (*P. pyrifolia* Nakai) according to their geographic distribution, and morphological and physiological traits (Teng and Tanabe, 2004).

Chinese white pear has been misassigned to *P.* x *bretschneideri* Rehder for a long time (Teng and Tanabe, 2004). Many researchers did not accept this classification because of the morphological differences between CWP and *P*. x *bretschneideri* (Kikuchi, 1946; Teng et al., 2002), and the many more similar morphological characteristics, such as leaf morphology and fruit texture (Pu and Wang, 1963), as well as peroxidase isozymic patterns (Lin and Shen, 1983), shared between CWP and CSP. Our previous studies (Teng et al., 2002; Bao et al., 2007; Jiang et al., 2015, 2016) and other researchers’ studies (Iketani et al., 2012; Song et al., 2014; Liu et al., 2015) using different DNA markers suggested that CWP was also genetically close to CSP. Meanwhile, JP cultivars may share the same germplasm as CSP based on similar morphological traits, especially fruit traits (Teng and Tanabe, 2004). Some researchers believed that these cultivars were domesticated from wild *P. pyrifolia* in Japan (Kikuchi, 1948), which seems to be supported by a few recent studies on the genetic relationships among Asian pears in which the majority of JPs did not cluster together with CSPs in dendrograms (Kimura et al., 2002; Nishio et al., 2016). However, other researchers suggested that JP cultivars might have been introduced from ancient China (Kajiura and Suzuki, 1980; Teng et al., 2002) because a flourishing sea route existed between Japan and the coastal areas of China. Thus, the so-called wild *P. pyrifolia* individuals in Japan were supposed to be escapees (Teng and Tanabe, 2004). The phenomenon of escapees is complicated in fruit trees owing to the existence of semi-wild or wild populations in nearby cultivated areas and to difficulties in distinguishing true wild types, escapees, and hybrids (Eliezer, 2013). This could be further complicated by gene flow and introgression from cultivated forms into wild relatives, as discovered in grape (Di Vecchi-Staraz et al., 2008), cultivated almond (Mohamed, 2004), and cultivated olive (Lavee and Zohary, 2011). Because of the genetic affinities of JP to pear cultivars from ZJ, based on multiple DNA markers, researchers proposed that this province might be the origin of JP cultivars (Teng et al., 2002; Bao et al., 2007; Jiang et al., 2016).

In the present study, phylogeographic analyses of cpDNA haplotypes suggested that CSP, CWP, and JP shared common cpDNA haplotypes (H14, H6, and H3) and that these haplotypes were distributed throughout geographic populations (**Figure 3A**). Even though the two major haplotype lineages (H14 and H6) displayed differential geographic distributions, they accounted for large proportions of all of the cultivar groups. Clusters *N*-8.1 and *N*-8.2 contained most of pear cultivars from CSP, CWP, and JP based on the Bayesian clustering of chloroplast DNA (**Figure 2B**). The results of STRUCTURE clustering inferred from nSSR data indicated that the CWP genotypes shared the major gene pools with CSP genotypes (**Figure 1A**), AMOVA analyses showed that there were very low level of genetic variations partitioned among cultivated groups of *P.pyrifolia* (2.49 and 3.61% for nSSR and cpDNA, respectively), which was supported by Iketani et al. (2012), inferring that they had a close genetic relationship, and supporting our previous hypothesis that CWP from northern China might be an ecotype of CSP (Bao et al., 2007). Nevertheless, STRUCTURE clustering clearly showed that the genetic structure of JP cultivars was distinctively different from those of CSP and CWP cultivars in China (**Figure 1A**), which is consistent with the previous studies where most JPs could not be clustered together with CSP cultivars in the dendrograms (Bao et al., 2007; Iketani et al., 2012; Nishio et al., 2016). The distinctiveness between the JP cultivar group and the CSP/CWP cultivar group, based on nuclear markers, might be explained by the limited primitive genetic backgrounds in the development of JP (Iketani et al., 2012), which was also supported by JP populations having the highest percentages of pure/non-admixed genotypes (**Figure 1B**). In addition, the dominant “green” gene pool in JPs was shared with some cultivars from ZJ and FJ in China (**Figure 1B**). Higher similarities were detected in maternally inherited cpDNA haplotypes of pear cultivars from both Japan and the coastal areas of China, which indicated that JP seeds could have been introduced from China through human activity. Thus, it was inferred that CSP and JP could be derived from the same progenitor of *P. pyrifolia* in China.

Because UP cultivars have different morphological traits than the cultivated *P. pyrifolia*, they have generally been considered as directly derived from the WUP of northeastern China (Pu and Wang, 1963; Teng and Tanabe, 2004), although a few studies revealed levels of genetic differentiation between UP cultivars and WUP (Wuyun et al., 2013; Nishio et al., 2016). In this study, 22 UP cultivars and 18 WUP were collected mainly from northeastern China for the genetic analyses. The cpDNA analysis determined that UPs share the common cpDNA haplotypes (H14 and H6) with cultivated *P. pyrifolia*: CSP, CWP, and JP (**Figure 3A**), and most of UPs and cultivars of CSP and CWP, especially those from southwest populations, were grouped in cluster *N*-8.1 in the Bayesian clustering of chloroplast DNA (**Figure 2B**). The dominant haplotypes of WUP were not detected in UP cultivar accessions, and two common haplotypes of UP did not occur in WUP accessions. Although both UP and WUP accessions were dominated by the same gene pool, as inferred from nSSR markers (**Figure 1A**), a high proportion of admixed genotypes were detected in UP accessions. Additionally, the two major gene pools (**Figure 1A**) of CSP and CWP also existed in UP accessions, while all of the WUP individuals were dominated by a single gene pool. And the accessions of WUP were mostly included in cluster *N*-8.8, separated from most of the cultivated Asian pears (**Figure 2B**). Thus, UP cultivars could not be directly domesticated from the WUP, and *P. pyrifolia* cultivars might be involved in their origin. This supports the findings of Jiang et al. (2016) and Yu et al. (2016) in which retrotransposon markers were used to determine that the major gene pools in UP cultivars came from cultivated *P. pyrifolia*. However, commercial UP cultivars generally have small fruit with poor quality (Kikuchi, 1946). In UP breeding programs, local UP cultivars should be further crossed with cultivated *P. pyrifolia* to gain a better fruit quality and maintain their cold tolerance. In addition, a higher level of genetic differentiation between UP and WUP was detected in cpDNA (*F*_ST_ = 0.5739) than in nSSRs (*F*_ST_ = 0.1031). By comparing Bayesian clustering based on cpDNA variations and nSSR STRUCTURE of the Asian pear accessions, we hypothesized that cultivars of *P. pyrifolia* might be the maternal parents of UP cultivars and that WUP might be the paternal parents. Thus, the UP cultivars included in this study might be derived from the interspecific hybridization of cultivated *P. pyrifolia* with local WUP. In addition, based on the sequence divergences of low-copy nuclear genes (such as *LFY*2) among *Pyrus* species, Zheng et al. (2014) proposed that occidental species might be the paternal parents of some UPs (i.e., ‘Ruanerli’). However, fewer UP cultivars were used in this study. Thus, the origin of UPs in East Asia might be more complex. In the future, different nuclear markers and more UP accessions of different geographic origin will be required to confirm their origins.

### Putative Origin Center and Dissemination Routes of Cultivated *P. pyrifolia*

Compared with annual crops, fewer studies have provided analyses of the origin and domestication of fruit trees (Eliezer, 2013). One of the best known is the study of Zohary and Spiegel-Roy (1975) in which they clarified the evolutionary history of four classical Old World fruits (olive, grape, date, and fig) using “fossil” evidence and morphological clues from living plants and their wild relatives. Recent studies on the origins of apple (*M. domestica*), based on molecular markers, identified the principal progenitor (*M. sieversii*) and secondary contributor (*M. sylvestris*) of modern apples by exploring genetic variations between the domesticated apple and its wild relatives (Velasco et al., 2010; Cornille et al., 2012). Even though the genome of CWP was published by Wu et al. (2013), the origin and evolutionary history of pear cultivars native to East Asia are still obscure and have not been well illuminated. This is mainly because of unavailable wild populations, especially for cultivated *P. pyrifolia*, which is considered the most important germplasm of Asian pear cultivars owing to its higher commercial value and wide cultivation. For fruit trees, the invention of vegetative propagation based on grafting or cutting revolutionized their domestication processes. These practices were necessary to fix the selected traits discovered in the wild and may have shortened the evolutionary distance between modern fruit cultivars and their wild progenitors (Zohary and Spiegel-Roy, 1975; Eliezer, 2013). The cultivation of Asian pears has been recorded from antiquity (Pu and Wang, 1963), when grafting had already become a well-established technique (Mudge et al., 2009). Thus, although no wild *P. pyrifolia* individuals were included in this study, the sampled local cultivars of different geographic origin could provide significant clues to the evolutionary history of cultivated Asian pears.

In this study, 401 cultivated *P. pyrifolia*, including seven geographic populations of CSP from southern China, five geographic populations of CWP from northern China, and two geographic populations of JP from across Japan were used in the genetic analyses (Supplementary Table S1). A greater level of genetic diversity was detected in the geographic populations of CSP from southern China compared with geographic populations of CWP and JP from northern China and Japan based on both nSSRs and cpDNA haplotypes (**Tables 1, 2**). This suggested that CSP accessions possessed the highest polymorphism level and might have contributed to the development of the CWP and JP. Furthermore, the high level of genetic diversity and the greatest number of cpDNA haplotypes were detected in the southwestern SC and YN population of CSP, suggesting that the southwest might be the origin center of cultivated *P. pyrifolia*. In contrast, a relative lower genetic diversity level and fewer cpDNA haplotypes were discovered in the two geographic populations of JP, indicating that they might be peripheral populations of cultivated *P. pyrifolia*.

**FIGURE 4:**
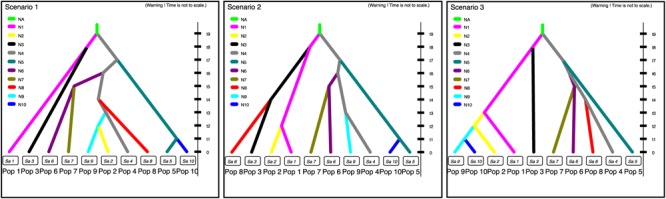
Three putative dissemination routes of cultivated *P. pyrifolia* in East Asia. Scenario 1 assumes that cultivated *P. pyrifolia* from southwestern regions (SC and YN) and Middle-Yangtze valley areas (HHJ) were diverged firstly, and then further spread from HHJ to southern (FJ and GG), eastern (AJ and ZJ) and northern areas (SS, GQ, SHH, and JL); Scenario 2 assumes that cultivated *P. pyrifolia* in northwestern (GQ) and southern areas (FJ and GG) were directly disseminated from southwestern populations (SC and YN); Scenario 3 assumes that cultivated *P. pyrifolia* from northwestern population (GQ) were initially disseminated from southwestern populations (SC and YN), from which cultivars were further spread to northern areas (SS, SHH and JL). Pop 1, YN+SC; Pop 2, GQ; Pop 3, GZ; Pop 4, HHJ; Pop 5, AJ; Pop 6, ZJ; Pop 7, JPa+JPb; Pop 8, FJ+GG; Pop 9, SS; and Pop 10, SHH+JL. The three scenarios represent the three historical models constructed based on approximate Bayesian computation, describing how the sampled populations are connected to their common ancestor. N1, N2, N3, N4, N5, N6, N7, N8, N9, and N10 represent the effective population sizes of Pop 1, Pop 2, Pop 3, Pop 4, Pop 5, Pop 6, Pop 7, Pop 8, Pop 9, and Pop 10, respectively. NA corresponds to a discrete change in the effective population size. The time is set as t9 > t8 > t7 > t6 > t5 > t4 > t3 > t2 > t1.

In contrast to nuclear DNA, which provided information on the current genetic structures, cpDNA, with a lower variation rate, could reveal the evolutionary histories of plants. The putative dissemination routes of cultivated *P. pyrifolia* were estimated from cpDNA haplotypes using approximate Bayesian computation (**Figure 4**), which suggested that cultivated *P. pyrifolia* might have been initially domesticated from wild *P. pyrifolia* in the southwest origin center. Then, cultivated pears disseminated across East Asia with the migration of people. Some of the cultivars spread from the original center to its eastern neighbor (i.e., GZ), while others spread to the Middle Yangtze River Valley (i.e., HHJ), then further to southern (ie., FJ and GG) and eastern China (ie., AJ and ZJ), as well as northern regions (i.e., SHH, Shanxi, and Liaoning Provinces). This indicated that cultivars from the Middle Yangtze River Valley areas might play pivotal roles in the southward, eastward and northward spread of cultivated *P. pyrifolia*, possibly resulting from the long historical commercial and cultural exchanges between Middle–Lower Yangtze Valley and North China. The composition of cpDNA haplotypes in geographical populations has been changed during the dissemination of cultivated *P. pyrifolia* possibly through conscious or unconscious selection, which may be one of the reasons that an increase of genetic differentiation based on cpDNA haplotype frequency was detected between populations from southwestern and populations from other regions (Supplementary Table S9). However, some rare haplotypes identified in few populations were not detected in cultivars from the putative origin center, such as haplotype H13, identified as the dominant and ancestral haplotype of oriental pears (Zheng et al., 2014), was only detected in a few cultivars scattered in northwestern (GQ) and southern (FJ and GG) populations that had higher numbers of unique nSSR alleles (**Table 1**). Cultivars containing these haplotypes might be involved with the interspecific hybridization between cultivated *P. pyrifolia* and local wild *Pyrus* species, rather than pollination of cultivated pears by local wild species, since chloroplast DNA was maternally inherited in Rosaceae and could be transmitted only by seeds.

Moreover, geographical population GQ were dominated by the two gene pools that had a relatively high number of unique alleles (8), might be explained by separate regional cultivar development. By analyzing the putative dissemination route of cultivated *P. pyrifolia*, the primitive cultivated pears from population GQ might have spread from the neighboring Shanxi Province, as described by Chang et al. (2017), along the ancient Silk Road (Teng et al., 2001). Comparatively, cultivars preserved in the mountain and hill areas of southwestern China might be more ancient types and should have the highest conservation priority.

### Comparison of Nuclear DNA- and cpDNA-Based Genetic Structures

Vegetative propagation based on grafting or cutting has undoubtedly revolutionized the domestication and breeding process of perennial trees, allowing the maintenance and spread of desirable lines despite self-incompatibility and a long juvenile phase (Janick, 2005). Because of the spread of grafting, “instant domestication” has been considered in the domestication of apple (Robinson et al., 2001). Under such circumstances, apple individuals with different geographic origins might be grafted and chosen from local wild types. Thus, the parental contributions in the origin of modern apple cultivars could be quite diverse (Robinson et al., 2001). Nuclear DNA is inherited parentally, and chloroplast DNA is inherited maternally in Rosaceae (Hu et al., 2008). The congruency of the nuclear DNA- and cpDNA-based genetic structures in the geographic populations might be attributed to the extensive practice of clonal propagation during the domestication of fruit trees, but the incongruence between them would indicate an intensive influence of cross-pollination (Nikiforova et al., 2013). The combination of nuclear SSR and chloroplast DNA has provided new insight into the origin of European pear cultivars (Ferradini et al., 2017). Therefore, the genetic comparison of parental inherited nuclear DNA markers and maternal inherited cpDNA in this study might generate meaningful results for the domestication of cultivated Asian pears.

In the present study, according to the gene pools of the current pear cultivars detected from nSSRs using Bayesian statistical inferences, three genetic groups were revealed for cultivated *P. pyrifolia*: (a) JPs; (b) cultivars from South-Central China northward to northeastern China, covering the main pear production area in China; (c) cultivars from southwestern China to southeastern China including Yunnan, Guizhou, Guangdong, Guangxi, and Fujian Provinces (**Figure 1B**), of which cultivars from South-Central China northward to northeastern China were also largely grouped in one cluster (*N*-8.2) based on Bayesian clustering results of cpDNA data and were dominated by the same cpDNA haplotype (H6) (**Figures 2B, 3A**). The congruency of the nuclear DNA- and cpDNA-based genetic structures in the geographic populations from these areas might be attributed to the extensive practice of clonal propagation during the spread of cultivated pears form Middle–Lower Yangtze River areas to the North China Plain. Low levels of genetic differentiation were also detected among populations within these areas based on both nSSRs and cpDNA (Supplementary Table S9). Based on the genetic groups of cultivated *P. pyrifolia* described above, the following three major groups for cultivated *P. pyrifolia* were suggested: Japanese, Central South-North China and Southwest to Southeast China.

More genetic clusters were revealed by Bayesian clustering of chloroplast DNA by comparison to genetic groups identified from nSSRs using Bayesian statistical inferences (**Figures 1A, 2A**). This is possibly due to some rare cpDNA haplotypes specific to certain geographic populations, since most of the cultivated pears were grouped in the first four clusters (Supplementary Table S5). However, most of cultivars from southernmost populations (i.e., GG and FJ) shared the major gene pools identified from nSSRs with cultivars from southwestern populations (i.e., SC, YN, and GZ), but they were dominated by different haplotypes (**Figure 3B**) and separately grouped in different clusters base on the Bayesian clustering of cpDNA (**Figure 2A**). A higher level of genetic differentiation was also detected from cpDNA than that from nSSRs between southwestern populations and southernmost populations. Because pear is a temperate wood species and needs low temperature exposure to break bud dormancy (Liu et al., 2012), cultivars from southern China (i.e., GG and FJ) may have become tolerant to warm temperatures in winter through conscious or unconscious selection (Eliezer, 2013), and most of cultivars carrying that haplotype were maintained during the selections. The differentiated genetic structures among geographic populations from different regions used in our study might reflect the synergistic effects of ecogeographical factors and human selection during cultivar improvement and spread. Diversity may have been restored to cultivars through gene introgression from wild types used as stocks when the cultivated clones were grown in new environments (Zohary and Spiegel-Roy, 1975).

Gene introgression from wild relatives has been detected in cultivated pears by some studies, indicating that it may contribute to the genetic diversification of current pear cultivars of different geographic origins (Zheng et al., 2014; Jiang et al., 2016). The introgression could be facilitated by self-incompatibility and cultural practices, such as selection from open-pollinated seeds (Nikiforova et al., 2013). For example, *P. pashia* may be involved in the development of cultivated *P. pyrifolia* in southwestern China (Zheng et al., 2014; Jiang et al., 2016) and wild *P. ussuriensis* may be involved in the evolution of CWP in northern China (Jiang et al., 2016). Although wild *Pyrus* species bear fruit with poor quality, they could contribute to disease resistance and to the adaptability of cultivated pears. High proportions of admixture genotypes based on paternal nSSRs detected in geographical populations SC, HHJ and GQ might be due to intensive introgression from local wild relatives. Therefore, samples of local wild species should be collected and genotyped for comparison with local cultivars to determine if wild relatives were involved in the development of local pear cultivars.

In summary, a high level of genetic diversity was detected in Asian pear cultivars, suggesting an increase in the level of genetic diversification that was represented by the Asian pears used in this study. AMOVA analyses based on both cpDNA fragments and genome-covering nSSR loci demonstrated close relationships among the four major pear cultivar groups according to relatively lower genetic variations partitioned among groups, but a higher level of genetic differentiation was revealed between UP and WUP. Phylogeographical analyses of cpDNA haplotypes indicated that CSP, CWP, and JP could be derived from the same progenitor of *P. pyrifolia* in China, and cultivars of *P. pyrifolia* might be the maternal parent of cultivated UPs. Southwestern populations including SC and YN, having a greater genotypic diversity, might be the origin center of cultivated *P. pyrifolia*. The putative dissemination route of cultivated *P. pyrifolia* indicated that cultivars from the Middle Yangtze River Valley might play important roles in the southward, eastward, and northward spread of cultivated *P. pyrifolia*. Based on the structural clustering of nSSRs, cultivated *P. pyrifolia* could be divided into the following major groups: Japanese, Central South-North China, and Southwest to Southeast China.

## Author Contributions

YT, XZ, and XY designed the experiments. XY, XZ, CH, GL, YC, and HH collected the plant materials. XY, XZ, CH, and PY performed the nSSR and cpDNA haplotype experiments. XY, XZ, SJ, YZ, and YT analyzed the data and drafted this manuscript.

## Conflict of Interest Statement

The authors declare that the research was conducted in the absence of any commercial or financial relationships that could be construed as a potential conflict of interest.

## References

[B1] Bacilieri R. Lacombe T. Le Cunff L. Di Vecchi-Staraz M. Laucou V. Genna B. (2013). Genetic structure in cultivated grapevines is linked to geography and human selection. 13:25. 10.1186/1471-2229-13-25 23394135 PMC3598926

[B2] Bailey L. H. (1917). *Pyrus*. 5 2865–2878.

[B3] Bao L. Chen K. Zhang D. Cao Y. Yamamoto T. Teng Y. (2007). Genetic diversity and similarity of pear (*Pyrus* L.) cultivars native to East Asia revealed by SSR (simple sequence repeat) markers. 54 959–971. 10.1631/jzus.B1300240 24711351 PMC3989149

[B4] Bao L. Chen K. Zhang D. Li X. Teng Y. (2008). An assessment of genetic variability and relationships within Asian pears based on AFLP (amplified fragment length polymorphism) markers. 116 374–380. 10.1007/s10722-009-9524-1

[B5] Cao Y. Tian L. Gao Y. Liu F. (2012). Genetic diversity of cultivated and wild Ussurian Pear (*Pyrus ussuriensis* Maxim.) in China evaluated with M13-tailed SSR markers. 59 9–17. 10.1007/s10722-011-9661-1

[B6] Challice J. S. Westwood M. N. (1973). Numerical taxonomic studies of the genus *Pyrus* using both chemical and botanical characters. 67 121–148. 10.1111/j.1095-8339.1973.tb01734.x

[B7] Chang Y. J. Cao Y. F. Zhang J. M. Tian L. M. Dong X. G. Zhang Y. (2017). Study on chloroplast DNA diversity of cultivated and wild pears (*Pyrus* L.) in Northern China. 13:44 10.1007/s11295-017-1126-z

[B8] Chen H. Song Y. Li L. Khan M. A. Li X. Korban S. S. (2014). Construction of a high-density simple sequence repeat consensus genetic map for pear (*Pyrus* spp.). 33 316–325. 10.1007/s11105-014-0745-x

[B9] Chen K. Abbott R. J. Milne R. I. Tian X. M. Liu J. (2008). Phylogeography of *Pinus tabulaeformis* Carr. (Pinaceae), a dominant species of coniferous forest in northern China. 17 4276–4288. 10.1111/j.1365-294X.2008.03911.x 19378405

[B10] Clement M. Posada D. Crandall K. A. (2000). TCS: a computer program to estimate gene genealogies. 9 1657–1659. 10.1046/j.1365-294x.2000.01020.x 11050560

[B11] Corander J. Gyllenberg M. Koski T. (2007). Random partition models and exchangeability for bayesian identification of population structure. 69 797–815. 10.1007/s11538-006-9161-1 17086368

[B12] Cornille A. Gladieux P. Smulders M. J. Roldán-Ruiz I. Laurens F. Le Cam B. (2012). New insight into the history of domesticated apple: secondary contribution of the European wild apple to the genome of cultivated varieties. 8:e1002703. 10.1371/journal.pgen.1002703 22589740 PMC3349737

[B13] Cornuet J. M. Pudlo P. Veyssier J. Dehne-Garcia A. Gautier M. Leblois R. (2014). DIYABC v2. 0: a software to make approximate Bayesian computation inferences about population history using single nucleotide polymorphism. DNA sequence and microsatellite data. 30 1187–1189. 10.1093/bioinformatics/btt763 24389659

[B14] Di Vecchi-Staraz M. Laucou V. Bruno G. Lacombe T. Gerber S. Bourse T. (2008). Low level of pollen-mediated gene flow from cultivated to wild grapevine: consequences for the evolution of the endangered subspecies *Vitis vinifera* L. subsp. *silvestris*. 100 66–75. 10.1093/jhered/esn084 18927474

[B15] Doyle J. J. (1987). A rapid DNA isolation procedure for small quantities of fresh leaf tissue. 19 11–15.

[B16] Earl D. A. (2012). STRUCTURE HARVESTER: a website and program for visualizing STRUCTURE output and implementing the Evanno method. 4 359–361. 10.1007/s12686-011-9548-7

[B17] Eliezer E. G. (2013). The evolution of fruit tree productivity: a review. 67 51–62. 10.1007/s12231-012-9219-y 23538880 PMC3606516

[B18] Evanno G. Regnaut S. Goudet J. (2005). Detecting the number of clusters of individuals using the software STRUCTURE: a simulation study. 14 2611–2620. 10.1111/j.1365-294X.2005.02553.x 15969739

[B19] Excoffier L. Lischer H. E. (2010). Arlequin suite ver 3.5: a new series of programs to perform population genetics analyses under Linux and Windows. 10 564–567. 10.1111/j.1755-0998.2010.02847.x 21565059

[B20] Ferradini N. Lancioni H. Torricelli R. Russi L. Dalla Ragione I. Cardinali I. (2017). Characterization and phylogenetic analysis of ancient Italian landraces of pear. 8:751. 10.3389/fpls.2017.00751 28539931 PMC5423897

[B21] Guilford P. Prakash S. Zhu J. Rikkerink E. Gardiner S. Bassett H. (1997). Microsatellites in *Malus* x *domestica* (apple): abundance, polymorphism and cultivar identification. 94 249–254. 10.1007/s001220050407

[B22] Heywood V. Casas A. Ford-Lloyd B. Kell S. Maxted N. (2007). Conservation and sustainable use of crop wild relatives. 121 245–255. 10.1016/j.agee.2006.12.014

[B23] Hu Y. Zhang Q. Rao G. Sodmergen G. (2008). Occurrence of plastids in the sperm cells of caprifoliaceae: biparental plastid inheritance in angiosperms is unilaterally derived from maternal inheritance. 49 958–968. 10.1093/pcp/pcn069 18448473

[B24] Huson D. H. Bryant D. (2006). Application of phylogenetic networks in evolutionary studies. 23 254–267. 10.1093/molbev/msj030 16221896

[B25] Iketani H. Katayama H. Uematsu C. Mase N. Sato Y. Yamamoto T. (2012). Genetic structure of East Asian cultivated pears (*Pyrus* spp.) and their reclassification in accordance with the nomenclature of cultivated plants. 298 1689–1700. 10.1007/s00606-012-0670-0

[B26] Jakobsson M. Rosenberg N. A. (2007). CLUMPP: a cluster matching and permutation program for dealing with label switching and multimodality in analysis of population structure. 23 1801–1806. 10.1093/bioinformatics/btm233 17485429

[B27] Janick J. (2005). The origins of fruits, fruit growing, and fruit breeding. 25 255–320. 10.1002/9780470650301.ch8

[B28] Janick J. (2000). The pear in history, literature, popular culture, and art. 596 41–52.

[B29] Jiang S. Zheng X. Yu P. Yue X. Ahmed M. Cai D. (2016). Primitive genepools of Asian pears and their complex hybrid origins inferred from fluorescent sequence-specific amplification polymorphism (SSAP) markers based on LTR retrotransposons. 11:e0149192. 10.1371/journal.pone.0149192 26871452 PMC4752223

[B30] Jiang S. Zong Y. Yue X. Postman J. Teng Y. Cai D. (2015). Prediction of retrotransposons and assessment of genetic variability based on developed retrotransposon-based insertion polymorphism (RBIP) markers in *Pyrus* L. 290 225–237. 10.1007/s00438-014-0914-5 25216935

[B31] Kajiura I. Suzuki S. (1980). Variations in fruit shapes of Japanese pear cultivars (*Pyrus serotina* Rehder var. *culta* Rehder). Geographic differentiation and changes. 30 309–328. 10.1270/jsbbs1951.30.309

[B32] Kikuchi A. (1946). Assessment of Chinese pear species and cultivars (in Japanese). 3 1–11.

[B33] Kikuchi A. (1948). *Horticulture of Fruit Trees (in Japanese)*. Tokyo: Yokendo 64–76.

[B34] Kimura T. Iketani H. Kotobuki K. Matsuta N. Ban Y. Hayashi T. (2003). Genetic characterization of pear varieties revealed by chloroplast DNA sequences. 78 241–247. 10.1080/14620316.2003.11511612

[B35] Kimura T. Shi Y. Z. Shoda M. Kotobuki K. Matsuta N. Hayashi T. (2002). Identification of Asian pear varieties by SSR analysis. 52 115–121. 10.1270/jsbbs.52.115

[B36] Kumar S. Kirk C. Deng C. Wiedow C. Knaebel M. Brewer L. (2017). Genotyping-by-sequencing of pear (*Pyrus* spp.) accessions unravels novel patterns of genetic diversity and selection footprints. 4:17015. 10.1038/hortres.2017.15 28451438 PMC5389204

[B37] Lavee S. Zohary D. (2011). The potential of genetic diversity and the effect of geographically isolated resources in olive breeding. 59 3–13. 10.1560/IJPS.59.1.3

[B38] Librado P. Rozas J. (2009). DnaSP v5: a software for comprehensive analysis of DNA polymorphism data. 25 1451–1452. 10.1093/bioinformatics/btp187 19346325

[B39] Lin B. Shen D. (1983). Studies on the germplasmic characteristics of *Pyrus* by use of isozymic patterns (in Chinese with English summary). 9 235–243.

[B40] Liu G. Li W. Zheng P. Tong X. Chen L. Liu D. (2012). Transcriptomic analysis of ‘Suli’ pear (*Pyrus pyrifolia* white pear group) buds during the dormancy by RNA-Seq. 13:700. 10.1186/1471-2164-13-700 23234335 PMC3562153

[B41] Liu J. Sun P. Zheng X. Potter D. Li K. Hu C. (2013). Genetic structure and phylogeography of *Pyrus pashia* L.(Rosaceae) in Yunnan Province, China, revealed by chloroplast DNA analyses. 9 433–441. 10.1007/s11295-012-0564-x

[B42] Liu Q. Song Y. Liu L. Zhang M. Sun J. Zhang S. (2015). Genetic diversity and population structure of pear (*Pyrus* spp.) collections revealed by a set of core genome-wide SSR markers. 11:128 10.1007/s11295-015-0953-z

[B43] Mohamed B. S. (2004). The contribution of *Prunus webbii* to almond evolution. 140 9–13.

[B44] Mudge K. Janick J. Scofield S. Goldschmidt E. E. (2009). 9. A history of grafting. 35:437 10.1002/9780470593776.ch9

[B45] Myles S. Boyko A. R. Owens C. L. Brown P. J. Grassi F. Aradhya M. K. (2011). Genetic structure and domestication history of the grape. 108 3530–3535. 10.1073/pnas.1009363108 21245334 PMC3048109

[B46] Nikiforova S. V. Cavalieri D. Velasco R. Goremykin V. (2013). Phylogenetic analysis of 47 chloroplast genomes clarifies the contribution of wild species to the domesticated apple maternal line. 30 1751–1760. 10.1093/molbev/mst092 23676769

[B47] Nishio S. Takada N. Saito T. Yamamoto T. Iketani H. (2016). Estimation of loss of genetic diversity in modern Japanese cultivars by comparison of diverse genetic resources in Asian pear (*Pyrus* spp.). 17:81. 10.1186/s12863-016-0380-7 27301575 PMC4908778

[B48] Peakall R. Smouse P. E. (2012). GenAIEx 6.5: genetic analysis in Excel. Population genetic software for teaching and research-an update. 28 2537–2539. 10.1093/bioinformatics/bts460 22820204 PMC3463245

[B49] Pons O. Petit R. (1996). Measuring and testing genetic differentiation with ordered versus unordered alleles. 144 1237–1245. 8913764 10.1093/genetics/144.3.1237 PMC1207615

[B50] Pritchard J. K. Stephens M. Donnelly P. (2000). Inference of population structure using multilocus genotype data. 155 945–959. 10.1093/genetics/155.2.945 PMC1461096 10835412

[B51] Pu F. Wang Y. (1963). *Pomology of China: Pears*. Shanghai: Shanghai Scientific and Technical Publishers.

[B52] Raymond M. Rousset F. (1995). GENEPOP (version 1.2): population genetics software for exact tests and ecumenicism. 86 248–249. 10.1093/oxfordjournals.jhered.a111573

[B53] Rehder A. (1915). “Synopsis of the Chinese species of *Pyrus*,” in New York, NY 225–241. 10.2307/20025539

[B54] Richards C. M. Volk G. M. Reilley A. A. Henk A. D. Lockwood D. R. Reeves P. A. (2009). Genetic diversity and population structure in *Malus sieversii*, a wild progenitor species of domesticated apple. 5 339–347. 10.1007/s11295-008-0190-9 22589740

[B55] Robinson J. Harris S. Juniper B. (2001). Taxonomy of the genus *Malus* Mill.(Rosaceae) with emphasis on the cultivated apple, *Malus domestica* Borkh. 226 35–58. 10.1007/s006060170072

[B56] Rosenberg N. A. (2004). DISTRUCT: a program for the graphical display of population structure. 4 137–138. 10.1046/j.1471-8286.2003.00566.x

[B57] Rubtsov G. (1944). Geographical distribution of the genus *Pyrus* and trends and factors in its evolution. 358–366. 10.1086/281206

[B58] Schuelke M. (2000). An economic method for the fluorescent labeling of PCR fragments. 18 233–234. 10.1038/72708 10657137

[B59] Shimura I. (1988). “Nashi (Pear),” in 36 ed. Heibonsha (Tokyo: Heibonsha) 354–372.

[B60] Song Y. Fan L. Chen H. Zhang M. Ma Q. Zhang S. (2014). Identifying genetic diversity and a preliminary core collection of *Pyrus pyrifolia* cultivars by a genome-wide set of SSR markers. 167 5–16. 10.1016/j.scienta.2013.12.005

[B61] Teng Y. Tanabe K. (2004). Reconsideration on the origin of cultivated pears native to East Asia. 634 175–182. 10.17660/ActaHortic.2004.634.21

[B62] Teng Y. Tanabe K. Tamura F. Itai A. (2001). Genetic relationships of pear cultivars in Xinjiang, China, as measured by RAPD markers. 76 771–779. 10.1080/14620316.2001.11511444

[B63] Teng Y. Tanabe K. Tamura F. Itai A. (2002). Genetic relationships of *Pyrus* species and cultivars native to East Asia revealed by randomly amplified polymorphic DNA markers. 127 262–270.

[B64] Thompson J. D. Gibson T. J. Plewniak F. Jeanmougin F. Higgins D. G. (1997). The CLUSTAL_X windows interface: flexible strategies for multiple sequence alignment aided by quality analysis tools. 25 4876–4882. 10.1093/nar/25.24.4876 9396791 PMC147148

[B65] Van Oosterhout C. Hutchinson W. F. Wills D. P. Shipley P. (2004). MICRO-CHECKER: software for identifying and correcting genotyping errors in microsatellite data. 4 535–538. 10.1111/j.1471-8286.2004.00684.x

[B66] Velasco R. Zharkikh A. Affourtit J. Dhingra A. Cestaro A. Kalyanaraman A. (2010). The genome of the domesticated apple (*Malus domestica* Borkh.). 42 833–839. 10.1038/ng.654 20802477

[B67] Wright S. (1978). Vol. 4 Chicago, IL: University of Chicago press

[B68] Wu J. Wang Z. Shi Z. Zhang S. Ming R. Zhu S. (2013). The genome of the pear (*Pyrus bretschneideri* Rehd.). 23 396–408. 10.1101/gr.144311.112 23149293 PMC3561880

[B69] Wuyun T. Ma T. Uematsu C. Katayama H. (2013). A phylogenetic network of wild Ussurian pears (*Pyrus ussuriensis* Maxim.) in China revealed by hypervariable regions of chloroplast DNA. 9 167–177. 10.1007/s11295-012-0544-1

[B70] Yamamoto T. Kimura T. Sawamura Y. Manabe T. Kotobuki K. Hayashi T. (2002a). Simple sequence repeats for genetic analysis in pear. 124 129–137. 10.1023/A:1015677505602

[B71] Yamamoto T. Kimura T. Shoda M. Imai T. Saito T. Sawamura Y. (2002b). Genetic linkage maps constructed by using an interspecific cross between Japanese and European pears. 106 9–18. 10.1007/s00122-002-0966-5 12582866

[B72] Yao L. Zheng X. Cai D. Gao Y. Wang K. Cao Y. (2010). Exploitation of Malus EST-SSRs and the utility in evaluation of genetic diversity in *Malus* and *Pyrus*. 57 841–851. 10.1007/s10722-009-9524-1

[B73] Yu P. Jiang S. Wang X. Bai S. Teng Y. (2016). Retrotransposon-based sequence-specific amplification polymorphism markers reveal that cultivated *Pyrus ussuriensis* originated from an interspecific hybridization. 81 264–272. 10.1371/journal.pone.0149192 26871452 PMC4752223

[B74] Yue X. Liu G. Zong Y. Teng Y. Cai D. (2014). Development of genic SSR markers from transcriptome sequencing of pear buds. 15 303–312. 10.1631/jzus.B1300240 24711351 PMC3989149

[B75] Zheng X. Cai D. Potter D. Postman J. Liu J. Teng Y. (2014). Phylogeny and evolutionary histories of *Pyrus* L. revealed by phylogenetic trees and networks based on data from multiple DNA sequences. 80 54–65. 10.1016/j.ympev.2014.07.009 25083939

[B76] Zohary D. Spiegel-Roy P. (1975). Beginning of fruit growing in the Old World. 187 319–327. 10.1126/science.187.4174.319 17814259

[B77] Zong Y. Sun P. Liu J. Yue X. Niu Q. Teng Y. (2014). Chloroplast DNA-based genetic diversity and phylogeography of *Pyrus betulifolia* (Rosaceae) in Northern China. 10 739–749. 10.1007/s11295-014-0718-0

