# Supplementary material for: Combined Analyses of Chloroplast DNA Haplotypes and Microsatellite Markers Reveal New Insights Into the Origin and Dissemination Route of Cultivated Pears Native to East Asia
Source: Front Plant Sci. 2018 May 7;9:591. doi: 10.3389/fpls.2018.00591 (PMC5949605; doi:10.3389/fpls.2018.00591)
Supplement: Supplementary file 2 [file Data_Sheet_1.docx]

Supplementary Material

Combined Analyses of Chloroplast DNA Haplotypes and Microsatellite Markers Reveal New Insights into the Origin and Dissemination Route of Cultivated Pears Native to East Asia

Xiaoyan Yue^1,2,3#^, Xiaoyan Zheng^4#^, Yu Zong^1,2,3^, Shuang Jiang^1,2,3^, Chunyun Hu^1,2,3^, Peiyuan Yu^1,2,3^, Guoqin Liu^5^, Yufen Cao^6^, Hongju Hu^7^, Yuanwen Teng^1,2,3^*

*** Correspondence:** Corresponding Author: ywteng@zju.edu.cn

# Supplementary Figures and Tables

## Supplementary Figures

##
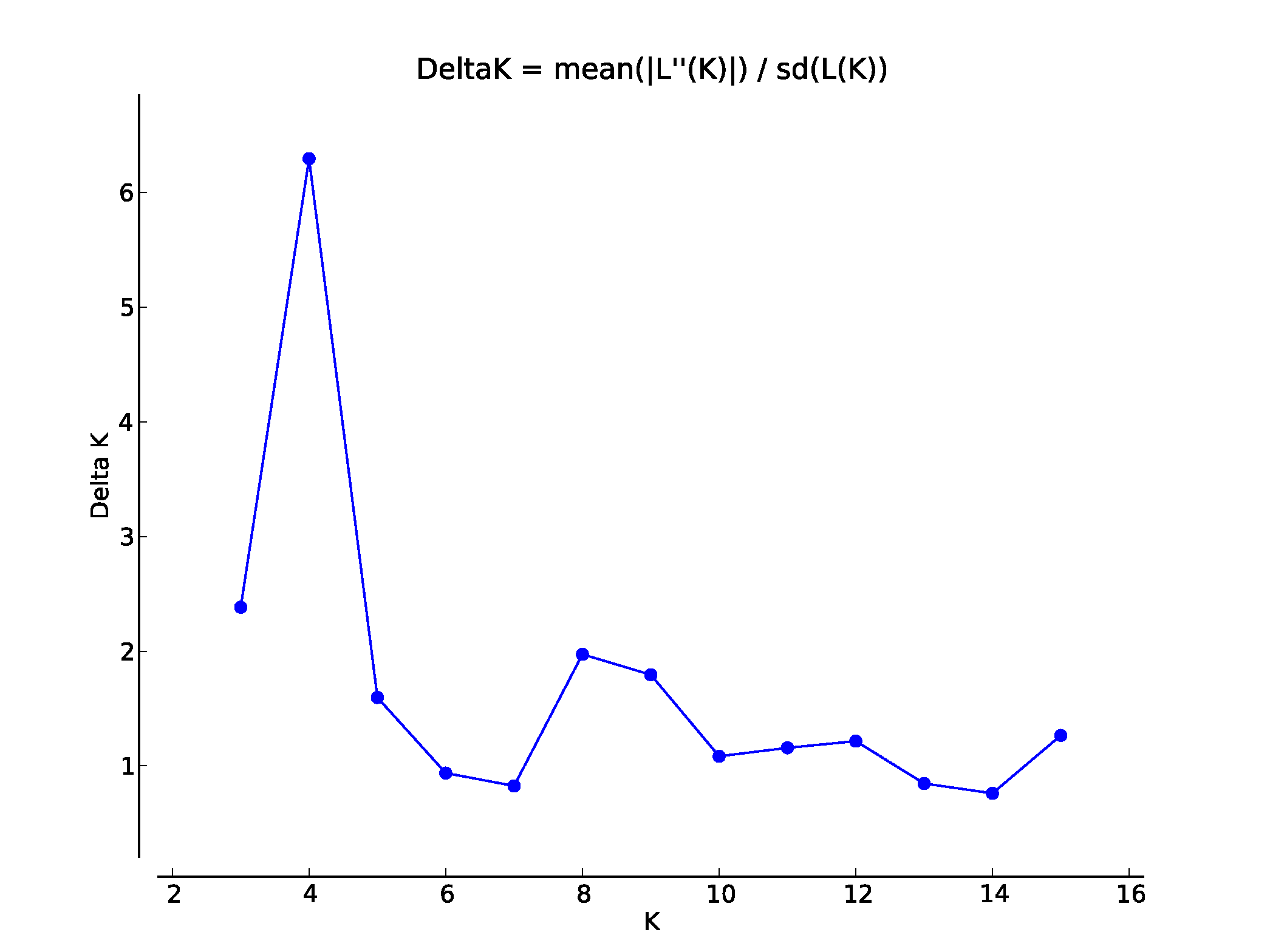
Supplementary figure S1. Modeling of group numbers for cultivated pears using STRUCTURE. Delta *K* was calculated in accordance with the method of Evanno et al. (2005).


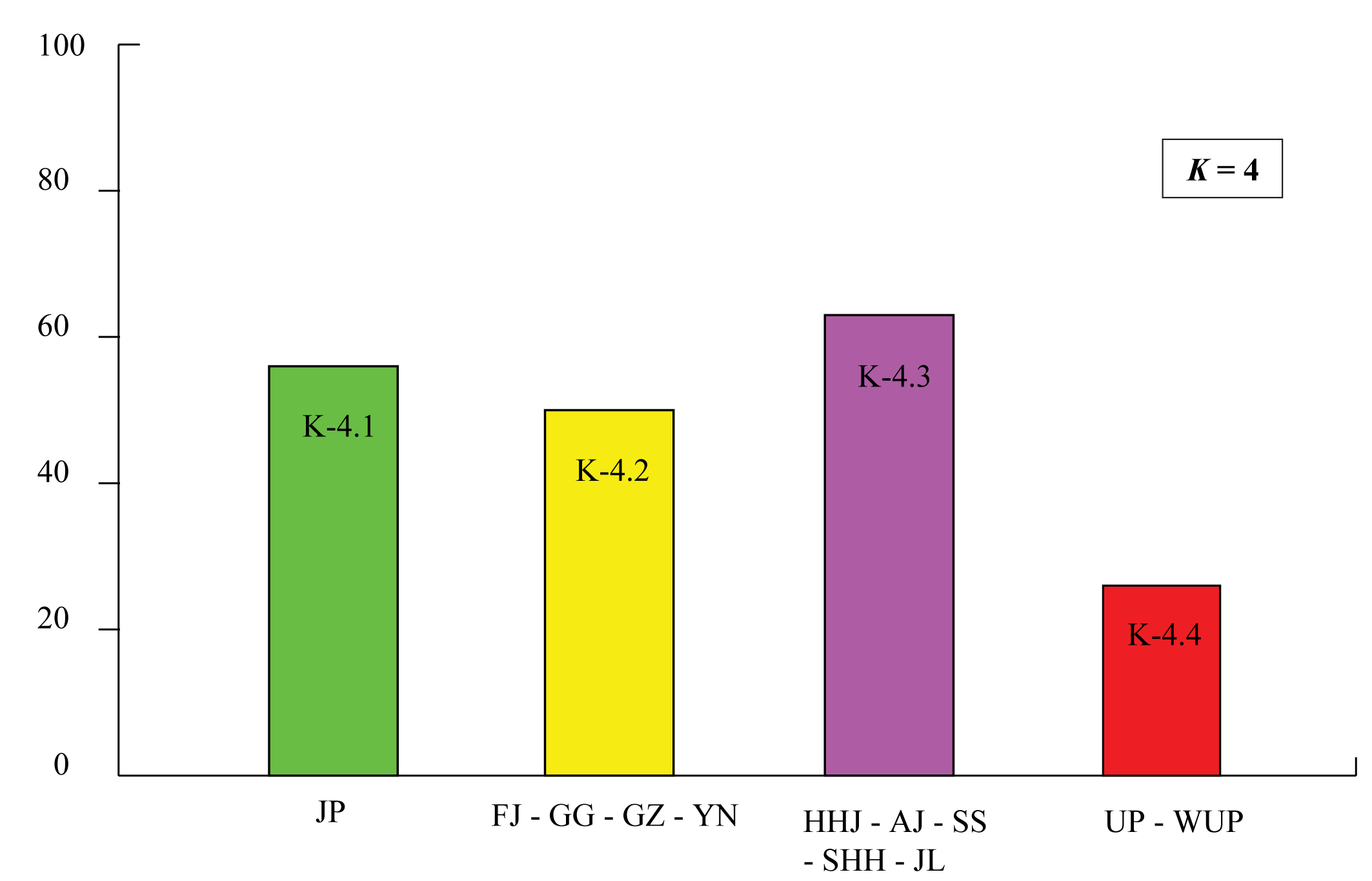


**Supplementary figure S2.** Characterization of genetic groups based on genetic compositions of geographic populations. The histograms represent the number of “non-admixed” genotypes. The color of each histogram corresponds to that of the gene pool in Figure 1.

##
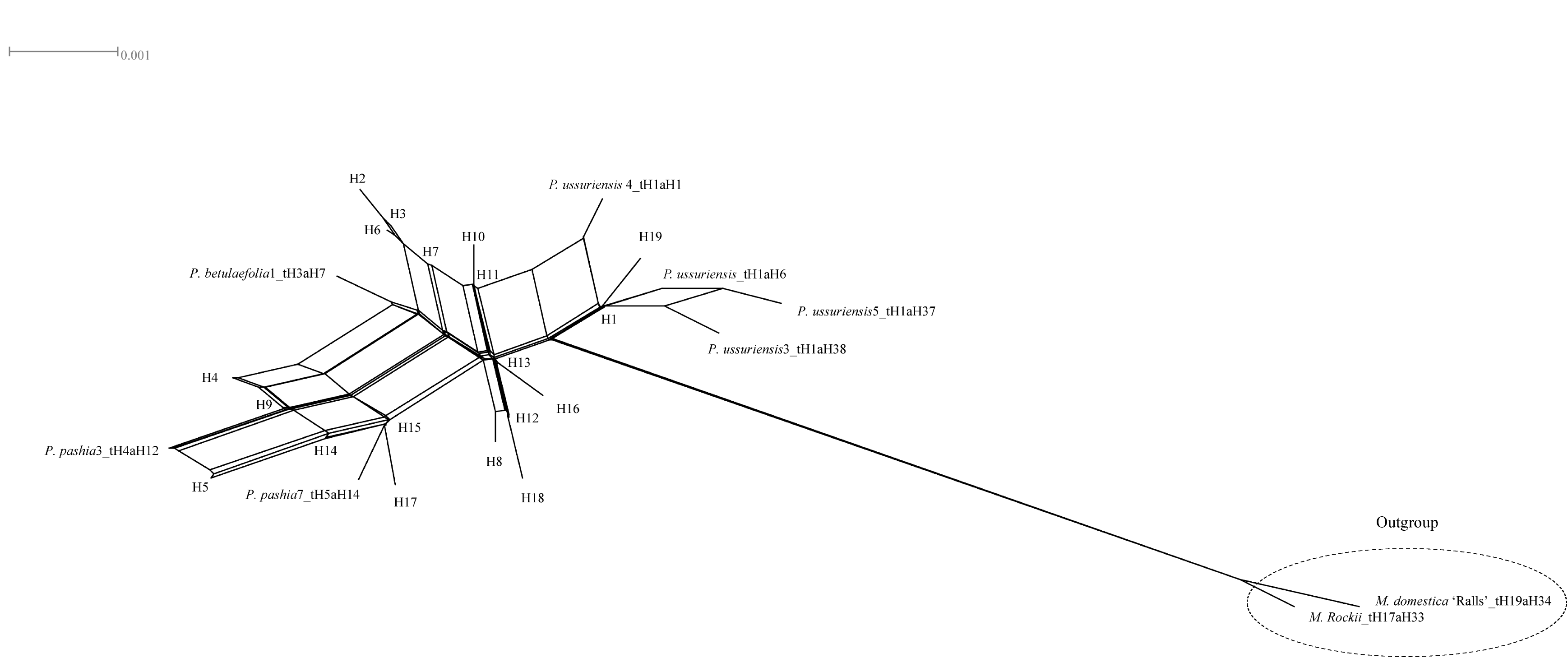


**Supplementary figure S3.** Neighbor-Net splits graph based on chloroplast DNA haplotype data.


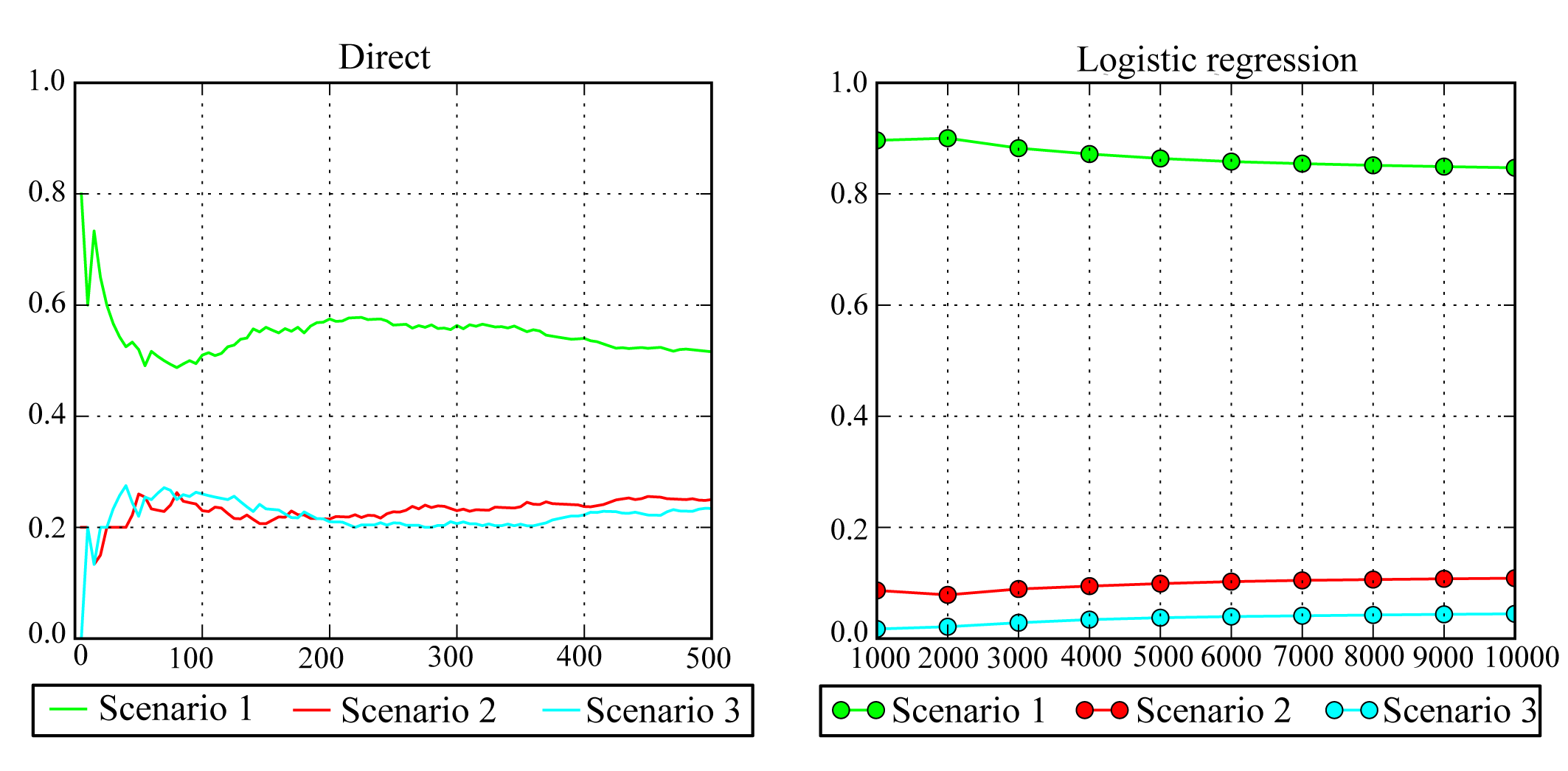


**Supplementary figure S4.** Direct (*left*) and logistic regression estimates (*right*) of posterior probabilities for three putative dissemination routes of cultivated *P. pyrifolia* in East Asia.


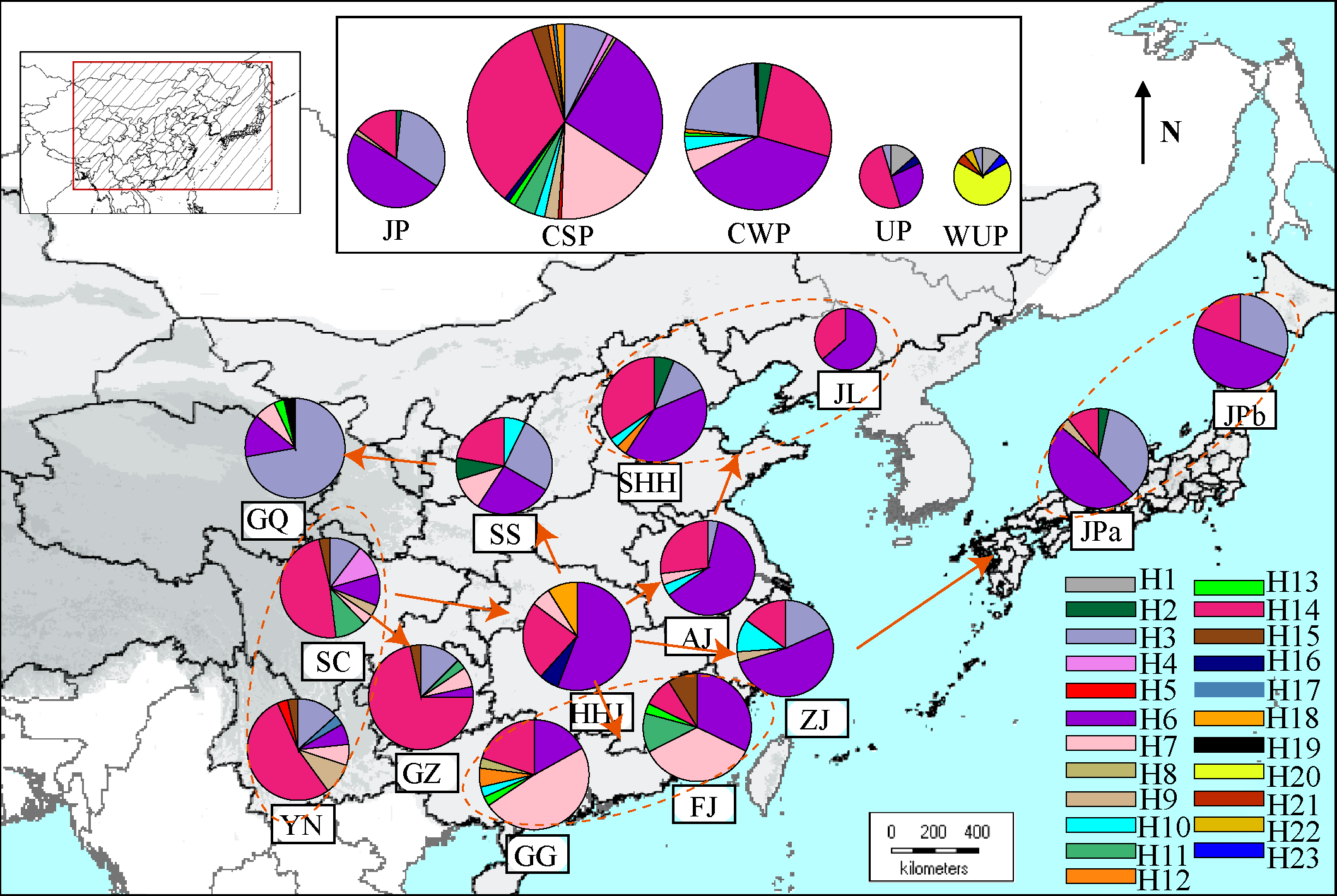


**Supplementary figure S5.** Map of putative dissemination routes for cultivated *P. pyrifolia* in East Asian. The arrows represent the evolutionary directions and the color of each haplotype and name of each geographic population correspond to those in Figure 1. The geographic populations in dashed circles correspond to the combined Pops in Figure 4.

## Supplementary Tables

# Supplementary table S1 Taxon information, chloroplast DNA haplotype, and nSSR genotype for each pear accession.

| Taxon | *No.* | Accession Name | Haplotype | tH | aH | SSR genotype | Geographic Origin | Leaf source | Population code |
| --- | --- | --- | --- | --- | --- | --- | --- | --- | --- |
| *Pyrus pyrifolia* | 55 | Asahiryu | H3 | tH3 | aH2 | Green | Honshu, Japan | TU | JPa |
| Japanese pear |  | Tsugaruao | H6 | tH4 | aH1 | Green | Honshu, Japan | TU | JPa |
|  |  | Shinchu | H6 | tH4 | aH1 | Green | Honshu, Japan | TU | JPa |
|  |  | Kozo | H6 | tH4 | aH1 | Green | Honshu, Japan | TU | JPa |
|  |  | Aoyagi | H6 | tH4 | aH1 | Green | Honshu, Japan | TU | JPa |
|  |  | Naganojisei | H3 | tH3 | aH2 | Green | Honshu, Japan | TU | JPa |
|  |  | Edoya | H3 | tH3 | aH2 | Green | Honshu, Japan | TU | JPa |
|  |  | Kawauchikoboku | H9 | tH4 | aH9 | Green | Honshu, Japan | TU | JPa |
|  |  | Kamenashi | H6 | tH4 | aH1 | Green | Honshu, Japan | TU | JPa |
|  |  | Awayuki | H3 | tH3 | aH2 | Green | Honshu, Japan | TU | JPa |
|  |  | Hatsushimo | H6 | tH4 | aH1 | Green | Honshu, Japan | TU | JPa |
|  |  | Akaho | H6 | tH4 | aH1 | Green | Honshu, Japan | TU | JPa |
|  |  | Chojuro | H6 | tH4 | aH1 | Green | Honshu, Japan | TU | JPa |
|  |  | Hakuteiryu | H2 | tH3 | aH1 | Green | Honshu, Japan | TU | JPa |
|  |  | Onba | H14 | tH5 | aH9 | Green | Honshu, Japan | TU | JPa |
|  |  | Nijisseiki | H6 | tH4 | aH1 | Green | Honshu, Japan | TU | JPa |
|  |  | Rokugatus | H3 | tH3 | aH2 | Green | Honshu, Japan | TU | JPa |
|  |  | Shinchi | H6 | tH4 | aH1 | Green | Honshu, Japan | TU | JPa |
|  |  | Konpeito | H3 | tH3 | aH2 | Green | Honshu, Japan | TU | JPa |
|  |  | Inugoroshi | H6 | tH4 | aH1 | Green | Honshu, Japan | TU | JPa |
|  |  | Tosajo | H3 | tH3 | aH2 | Green | Honshu, Japan | TU | JPa |
|  |  | Kunitomi | H6 | tH4 | aH1 | Purple | Honshu, Japan | TU | JPa |
|  |  | Okusankichi | H6 | tH4 | aH1 | Green | Shikoku, Japan | TU | JPb |
|  |  | Soranokawa | H6 | tH4 | aH1 | Green | Honshu, Japan | TU | JPa |
|  |  | Shoumyoujinashi | H3 | tH3 | aH2 | Green | Honshu, Japan | TU | JPa |
|  |  | Ookoga | H14 | tH5 | aH9 | Green | Honshu, Japan | TU | JPa |
|  |  | Shimoichikoboku | H3 | tH3 | aH2 | Admixed | Honshu, Japan | TU | JPa |
|  |  | Tsugaru | H6 | tH4 | aH1 | Green | Honshu, Japan | TU | JPa |
|  |  | Tsukutonashi | H3 | tH3 | aH2 | Green | Honshu, Japan | TU | JPa |
|  |  | Fukushima | H3 | tH3 | aH2 | Green | Japan | TU | JPb |
|  |  | Nekogoroshi | H3 | tH3 | aH2 | Green | Kyushu, Japan | TU | JPb |
|  |  | Geishun | H14 | tH5 | aH9 | Green | Japan | TU | JPb |
|  |  | Hondoensis | H14 | tH5 | aH9 | Admixed | Japan | TU | JPa |
|  |  | Tosan | H3 | tH3 | aH2 | Admixed | Shikoku, Japan | TU | JPb |
|  |  | Ichiharawase | H3 | tH3 | aH2 | Green | Shikoku, Japan | TU | JPb |
|  |  | Tosanashi | H6 | tH4 | aH1 | Green | Shikoku, Japan | TU | JPb |
|  |  | Tosanishiki | H6 | tH4 | aH1 | Green | Shikoku, Japan | TU | JPb |
|  |  | Ichiharawase | H6 | tH4 | aH1 | Green | Shikoku, Japan | TU | JPb |
|  |  | Umajirou | H6 | tH4 | aH1 | Green | Shikoku, Japan | TU | JPb |
|  |  | Imamuraaki | H6 | tH4 | aH1 | Green | Shikoku, Japan | TU | JPb |
|  |  | Kansaiichi | H14 | tH5 | aH9 | Green | Japan | TU | JPb |
|  |  | Shimokaburi | H14 | tH5 | aH9 | Green | Kyushu, Japan | TU | JPb |
|  |  | Hakataao | H14 | tH5 | aH9 | Green | Kyushu, Japan | TU | JPb |
|  |  | Kouchi6 | H14 | tH5 | aH9 | Admixed | Kyushu, Japan | TU | JPb |
|  |  | Kouchi7 | H3 | tH3 | aH2 | Green | Japan | TU | JPb |
|  |  | Yamanashi | H3 | tH3 | aH2 | Green | Shikoku, Japan | TU | JPb |
|  |  | kouchi28 | H6 | tH4 | aH1 | Green | Shikoku, Japan | TU | JPb |
|  |  | kouchi8 | H6 | tH4 | aH1 | Green | Shikoku, Japan | TU | JPb |
|  |  | kouchi5 | H3 | tH3 | aH2 | Admixed | Shikoku, Japan | TU | JPb |
|  |  | kouchi29 | H6 | tH4 | aH1 | Admixed | Shikoku, Japan | TU | JPb |
|  |  | kouchi25 | H6 | tH4 | aH1 | Admixed | Shikoku, Japan | TU | JPb |
|  |  | Kouchi24 | H6 | tH4 | aH1 | Green | Shikoku, Japan | TU | JPb |
|  |  | kouchi12 | H3 | tH3 | aH2 | Admixed | Shikoku, Japan | TU | JPb |
|  |  | kouchi11 | H6 | tH4 | aH1 | Green | Shikoku, Japan | TU | JPb |
|  |  | Babauchi | H6 | tH4 | aH1 | Admixed | Shikoku, Japan | TU | JPb |
| *P. pyrifolia* | 221 | Zaosanhua | H6 | tH4 | aH1 | Admixed | Zhejiang, China | CPGR | ZJ |
| Chinese sand pear | | Dahuangcha | H9 | tH4 | aH9 | Green | Zhejiang, China | WSGR | ZJ |
|  |  | Nuodao | H6 | tH4 | aH1 | Admixed | Zhejiang, China | WSGR | ZJ |
|  |  | Yandangxueli | H6 | tH4 | aH1 | Admixed | Zhejiang, China | WSGR | ZJ |
|  |  | Yunlv | H6 | tH4 | aH1 | Admixed | Zhejiang, China | WSGR | ZJ |
|  |  | Xiaohuangli | H6 | tH4 | aH1 | Admixed | Zhejiang, China | WSGR | ZJ |
|  |  | Daenli | H6 | tH4 | aH1 | Admixed | Zhejiang, China | WSGR | ZJ |
|  |  | Huangqieli | H14 | tH5 | aH9 | Green | Zhejiang, China | WSGR | ZJ |
|  |  | Pugua | H6 | tH4 | aH1 | Admixed | Zhejiang, China | WSGR | ZJ |
|  |  | Yuanli | H3 | tH3 | aH2 | Admixed | Zhejiang, China | WSGR | ZJ |
|  |  | Sanhua | H6 | tH4 | aH1 | Admixed | Zhejiang, China | WSGR | ZJ |
|  |  | Huahong | H14 | tH5 | aH9 | Admixed | Zhejiang, China | WSGR | ZJ |
|  |  | Rentouli | H10 | tH5 | aH1 | Admixed | Zhejiang, China | WSGR | ZJ |
|  |  | Zhenxiang | H3 | tH3 | aH2 | Admixed | Zhejiang, China | WSGR | ZJ |
|  |  | Zhuxinhuangli | H6 | tH4 | aH1 | Admixed | Zhejiang, China | WSGR | ZJ |
|  |  | Shengxianshali | H6 | tH4 | aH1 | Admixed | Zhejiang, China | WSGR | ZJ |
|  |  | Jiuzhong | H10 | tH5 | aH1 | Green | Zhejiang, China | WSGR | ZJ |
|  |  | Guihua | H3 | tH3 | aH2 | Admixed | Zhejiang, China | WSGR | ZJ |
|  |  | Anhuixueli | H14 | tH5 | aH9 | Admixed | Zhejiang, China | WSGR | ZJ |
|  |  | Yiwulizi | H6 | tH4 | aH1 | Purple | Zhejiang, China | WSGR | ZJ |
|  |  | Yanzhouxueli | H6 | tH4 | aH1 | Purple | Zhejiang, China | WSGR | ZJ |
|  |  | Huangjinli | H6 | tH4 | aH1 | Admixed | Zhejiang, China | WSGR | ZJ |
|  |  | Chisanhua | H3 | tH3 | aH2 | Admixed | Zhejiang, China | WSGR | ZJ |
|  |  | Meili | H3 | tH3 | aH2 | Admixed | Zhejiang, China | WSGR | ZJ |
|  |  | Huizhouxueli | H10 | tH5 | aH1 | Admixed | Zhejiang, China | WSGR | ZJ |
|  |  | Muguali | H14 | tH5 | aH9 | Admixed | Zhejiang, China | WSGR | ZJ |
|  |  | Liuyuexiao | H6 | tH4 | aH1 | Admixed | Zhejiang, China | WSGR | ZJ |
|  |  | Shandongli | H11 | tH5 | aH2 | Yellow | Fujian, China | Fujian, China | FJ |
|  |  | Baihuli | H14 | tH5 | aH9 | Purple | Fujian, China | Fujian, China | FJ |
|  |  | Damali | H7 | tH4 | aH2 | Purple | Fujian, China | Fujian, China | FJ |
|  |  | Tuli | H15 | tH5 | aH10 | Admixed | Fujian, China | Fujian, China | FJ |
|  |  | Aijiali | H11 | tH5 | aH2 | Admixed | Fujian, China | WSGR | FJ |
|  |  | Huaguli | H7 | tH4 | aH2 | Yellow | Fujian, China | Fujian, China | FJ |
|  |  | Huangxiao | H7 | tH4 | aH2 | Yellow | Fujian, China | Fujian, China | FJ |
|  |  | Maozali | H6 | tH4 | aH1 | Yellow | Fujian, China | Fujian, China | FJ |
|  |  | Longtanli | H6 | tH4 | aH1 | Admixed | Fujian, China | Fujian, China | FJ |
|  |  | Pinghexueli | H6 | tH4 | aH1 | Yellow | Fujian, China | Fujian, China | FJ |
|  |  | Qingpili | H11 | tH5 | aH2 | Yellow | Fujian, China | Fujian, China | FJ |
|  |  | Zaoshuli | H6 | tH4 | aH1 | Admixed | Fujian, China | Fujian, China | FJ |
|  |  | Dongmali | H7 | tH4 | aH2 | Yellow | Fujian, China | Fujian, China | FJ |
|  |  | Huangpizhuli | H7 | tH4 | aH2 | Yellow | Fujian, China | Fujian, China | FJ |
|  |  | Gaodili | H11 | tH5 | aH2 | Yellow | Fujian, China | Fujian, China | FJ |
|  |  | Chenhuali | H7 | tH4 | aH2 | Admixed | Fujian, China | Fujian, China | FJ |
|  |  | Shanzali | H7 | tH4 | aH2 | Admixed | Fujian, China | Fujian, China | FJ |
|  |  | Bayuexue | H7 | tH4 | aH2 | Yellow | Fujian, China | Fujian, China | FJ |
|  |  | Hanli | H15 | tH5 | aH10 | Admixed | Fujian, China | Fujian, China | FJ |
|  |  | Cuhuangli | H7 | tH4 | aH2 | Yellow | Fujian, China | Fujian, China | FJ |
|  |  | Xiandiaoli | H15 | tH5 | aH10 | Admixed | Fujian, China | Fujian, China | FJ |
|  |  | Liuyuexiao | H14 | tH5 | aH9 | Admixed | Fujian, China | Fujian, China | FJ |
|  |  | Qiyuehuang | H7 | tH4 | aH2 | Admixed | Fujian, China | Fujian, China | FJ |
|  |  | Renshoutingli | H7 | tH4 | aH2 | Admixed | Fujian, China | Fujian, China | FJ |
|  |  | Bingzili | H6 | tH4 | aH1 | Admixed | Fujian, China | CPGR | FJ |
|  |  | Qiubaisha | H6 | tH4 | aH1 | Admixed | Fujian, China | CPGR | FJ |
|  |  | Zongbaoli | H7 | tH4 | aH2 | Admixed | Fujian, China | CPGR | FJ |
|  |  | Mandingxueli | H6 | tH4 | aH1 | Admixed | Fujian, China | WSGR | FJ |
|  |  | Xihuaxueli | H6 | tH4 | aH1 | Admixed | Fujian, China | WSGR | FJ |
|  |  | Bannannvli | H6 | tH4 | aH1 | Green | Fujian, China | WSGR | FJ |
|  |  | Zhenghedaxueli | H14 | tH5 | aH9 | Admixed | Fujian, China | WSGR | FJ |
|  |  | Xihuapingtouli | H6 | tH4 | aH1 | Admixed | Fujian, China | WSGR | FJ |
|  |  | Qingpizhongli | H13 | tH5 | aH8 | Admixed | Fujian, China | CPGR | FJ |
|  |  | Huapili | H6 | tH4 | aH1 | Admixed | Fujian, China | WSGR | FJ |
|  |  | Fengkaihehuali | H7 | tH4 | aH2 | Admixed | Guangdong, China | WSGR | GG |
|  |  | Huiyangsuanli | H7 | tH4 | aH2 | Admixed | Guangdong, China | WSGR | GG |
|  |  | Huiyangxiangshuili | H7 | tH4 | aH2 | Yellow | Guangdong, China | WSGR | GG |
|  |  | Fengkaixinghuadayinli | H12 | tH5 | aH4 | Admixed | Guangdong, China | WSGR | GG |
|  |  | Fengkaiyeshenli | H7 | tH4 | aH2 | Admixed | Guangdong, China | WSGR | GG |
|  |  | Fengkaihuizhouli | H7 | tH4 | aH2 | Yellow | Guangdong, China | WSGR | GG |
|  |  | Huiyanghongli | H12 | tH5 | aH4 | Admixed | Guangdong, China | WSGR | GG |
|  |  | Jingxidongli | H6 | tH4 | aH1 | Admixed | Guangxi, China | Guangxi, China | GG |
|  |  | Daguotangli | H6 | tH4 | aH1 | Admixed | Guangxi, China | Guangxi, China | GG |
|  |  | Pohetangli | H7 | tH4 | aH2 | Yellow | Guangxi, China | Guangxi, China | GG |
|  |  | Daguoqingpi | H6 | tH4 | aH1 | Yellow | Guangxi, China | Guangxi, China | GG |
|  |  | Huangpitangli | H7 | tH4 | aH2 | Admixed | Guangxi, China | Guangxi, China | GG |
|  |  | Chengtuoli | H7 | tH4 | aH2 | Admixed | Guangxi, China | Guangxi, China | GG |
|  |  | Guanyangxueli | H6 | tH4 | aH1 | Admixed | Guangxi, China | Guangxi, China | GG |
|  |  | Qingshuili | H7 | tH4 | aH2 | Admixed | Guangxi, China | Guangxi, China | GG |
|  |  | Baipishuinanli | H7 | tH4 | aH2 | Admixed | Guangxi, China | Guangxi, China | GG |
|  |  | Zali | H7 | tH4 | aH2 | Admixed | Guangxi, China | Guangxi, China | GG |
|  |  | Matuoli | H7 | tH4 | aH2 | Admixed | Guangxi, China | Guangxi, China | GG |
|  |  | Cangwudashali | H14 | tH5 | aH9 | Admixed | Guangxi, China | CPGR | GG |
|  |  | Xiangjiaoli | H7 | tH4 | aH2 | Admixed | Guangxi, China | WSGR | GG |
|  |  | Hengxianmili | H7 | tH4 | aH2 | Yellow | Guangxi, China | Guangxi, China | GG |
|  |  | Guanyanghongpili | H14 | tH5 | aH9 | Admixed | Guangxi, China | WSGR | GG |
|  |  | Guanyangzaoheli | H6 | tH4 | aH1 | Admixed | Guangxi, China | WSGR | GG |
|  |  | Hengxianjinpaoli | H13 | tH5 | aH8 | Admixed | Guangxi, China | WSGR | GG |
|  |  | Hengxiansuanli | H8 | tH4 | aH4 | Admixed | Guangxi, China | WSGR | GG |
|  |  | Guangxili | H14 | tH5 | aH9 | Yellow | Guangxi, China | WSGR | GG |
|  |  | Jingxiqingpili | H6 | tH4 | aH1 | Admixed | Guangxi, China | WSGR | GG |
|  |  | Lipuxueli | H10 | tH5 | aH1 | Admixed | Guangxi, China | WSGR | GG |
|  |  | Liuchengxueli | H14 | tH5 | aH9 | Admixed | Guangxi, China | WSGR | GG |
|  |  | Sanmenjiangshali | H7 | tH4 | aH2 | Admixed | Guangxi, China | WSGR | GG |
|  |  | Si’anqingpili | H7 | tH4 | aH2 | Yellow | Guangxi, China | WSGR | GG |
|  |  | Beiliumili | H7 | tH4 | aH2 | Admixed | Guangxi, China | WSGR | GG |
|  |  | Guanyangcupitangli | H14 | tH5 | aH9 | Admixed | Guangxi, China | WSGR | GG |
|  |  | Guanyangdaqingpili | H14 | tH5 | aH9 | Admixed | Guangxi, China | WSGR | GG |
|  |  | Liuchengfengshanli | H14 | tH5 | aH9 | Admixed | Guangxi, China | WSGR | GG |
|  |  | Mopanli | H14 | tH5 | aH9 | Admixed | Guizhou, China | Guizhou, China | GZ |
|  |  | Piaobali | H14 | tH5 | aH9 | Yellow | Guizhou, China | Guizhou, China | GZ |
|  |  | Butangli | H14 | tH5 | aH9 | Admixed | Guizhou, China | Guizhou, China | GZ |
|  |  | Huahongli | H14 | tH5 | aH9 | Admixed | Guizhou, China | Guizhou, China | GZ |
|  |  | Dongli | H7 | tH4 | aH2 | Admixed | Guizhou, China | Guizhou, China | GZ |
|  |  | Xipihuangli | H14 | tH5 | aH9 | Yellow | Guizhou, China | Guizhou, China | GZ |
|  |  | Chengxiangli | H14 | tH5 | aH9 | Yellow | Guizhou, China | Guizhou, China | GZ |
|  |  | Shuiqingli | H3 | tH3 | aH2 | Yellow | Guizhou, China | Guizhou, China | GZ |
|  |  | Cili | H3 | tH3 | aH2 | Yellow | Guizhou, China | Guizhou, China | GZ |
|  |  | Damali | H7 | tH4 | aH2 | Yellow | Guizhou, China | Guizhou, China | GZ |
|  |  | Suanqingli | H3 | tH3 | aH2 | Admixed | Guizhou, China | Guizhou, China | GZ |
|  |  | Chengtuoli | H14 | tH5 | aH9 | Yellow | Guizhou, China | Guizhou, China | GZ |
|  |  | Semali | H14 | tH5 | aH9 | Yellow | Guizhou, China | Guizhou, China | GZ |
|  |  | Pidaili | H14 | tH5 | aH9 | Yellow | Guizhou, China | Guizhou, China | GZ |
|  |  | Xiangmianli | H14 | tH5 | aH9 | Yellow | Guizhou, China | Guizhou, China | GZ |
|  |  | Zhuibajiuyueli | H14 | tH5 | aH9 | Yellow | Guizhou, China | Guizhou, China | GZ |
|  |  | Liuyueli | H14 | tH5 | aH9 | Yellow | Guizhou, China | Guizhou, China | GZ |
|  |  | Mapijiuyueli | H14 | tH5 | aH9 | Yellow | Guizhou, China | Guizhou, China | GZ |
|  |  | Baipijiuyueli | H14 | tH5 | aH9 | Yellow | Guizhou, China | Guizhou, China | GZ |
|  |  | Xiaohuangli | H14 | tH5 | aH9 | Yellow | Guizhou, China | Guizhou, China | GZ |
|  |  | Muguali | H6 | tH4 | aH1 | Yellow | Guizhou, China | Guizhou, China | GZ |
|  |  | Huangshuili | H14 | tH5 | aH9 | Yellow | Guizhou, China | Guizhou, China | GZ |
|  |  | Fanli | H11 | tH5 | aH2 | Yellow | Guizhou, China | Guizhou, China | GZ |
|  |  | Hongfenli | H15 | tH5 | aH10 | Admixed | Guizhou, China | CPGR | GZ |
|  |  | Huishuijingai | H14 | tH5 | aH9 | Yellow | Guizhou, China | CPGR | GZ |
|  |  | Weiningdahuangli | H14 | tH5 | aH9 | Yellow | Guizhou, China | CPGR | GZ |
|  |  | Xingyihaizi | H14 | tH5 | aH9 | Yellow | Guizhou, China | CPGR | GZ |
|  |  | Meitanjingai | H14 | tH5 | aH9 | Admixed | Guizhou, China | WSGR | GZ |
|  |  | Baiwali | H14 | tH5 | aH9 | Admixed | Guizhou, China | WSGR | GZ |
|  |  | Weiningzaobaili | H14 | tH5 | aH9 | Admixed | Guizhou, China | WSGR | GZ |
|  |  | Huazhali | H14 | tH5 | aH9 | Yellow | Guizhou, China | WSGR | GZ |
|  |  | Wuli | H3 | tH3 | aH2 | Admixed | Guizhou, China | CPGR | GZ |
|  |  | Huangpishui | H6 | tH4 | aH1 | Admixed | Yunnan, China | WSGR | YN |
|  |  | Baozhuli | H14 | tH5 | aH9 | Admixed | Yunnan, China | CPGR | YN |
|  |  | Fuyuanhuangli | H14 | tH5 | aH9 | Yellow | Yunnan, China | CPGR | YN |
|  |  | Haidong | H14 | tH5 | aH9 | Admixed | Yunnan, China | CPGR | YN |
|  |  | Kunmingma | H9 | tH4 | aH9 | Admixed | Yunnan, China | CPGR | YN |
|  |  | Wuduhuobali | H14 | tH5 | aH9 | Yellow | Yunnan, China | CPGR | YN |
|  |  | Dabaozhu | H14 | tH5 | aH9 | Admixed | Yunnan, China | CPGR | YN |
|  |  | Lusha | H14 | tH5 | aH9 | Yellow | Yunnan, China | CPGR | YN |
|  |  | Niutou | H3 | tH3 | aH2 | Admixed | Yunnan, China | CPGR | YN |
|  |  | Huobali | H5 | tH5 | aH12 | Admixed | Yunnan, China | CPGR | YN |
|  |  | Sangmei | H7 | tH4 | aH2 | Admixed | Yunnan, China | CPGR | YN |
|  |  | Shuaili | H9 | tH4 | aH9 | Admixed | Yunnan, China | CPGR | YN |
|  |  | Xibaqingshui | H14 | tH5 | aH9 | Yellow | Yunnan, China | CPGR | YN |
|  |  | Miduxiaohongli | H14 | tH5 | aH9 | Admixed | Yunnan, China | WSGR | YN |
|  |  | Dalinaili | H14 | tH5 | aH9 | Admixed | Yunnan, China | WSGR | YN |
|  |  | Dalijitui | H14 | tH5 | aH9 | Yellow | Yunnan, China | WSGR | YN |
|  |  | Lijianghuangsuanli | H3 | tH3 | aH2 | Yellow | Yunnan, China | WSGR | YN |
|  |  | Miduxiaomianli | H17 | tH7 | aH10 | Yellow | Yunnan, China | WSGR | YN |
|  |  | Kunmingxiaoheshan | H15 | tH5 | aH10 | Admixed | Yunnan, China | WSGR | YN |
|  |  | Miduhuoba | H14 | tH5 | aH9 | Yellow | Yunnan, China | WSGR | YN |
|  |  | Lijiangmianli | H14 | tH5 | aH9 | Admixed | Yunnan, China | WSGR | YN |
|  |  | Daliyanzhi | H3 | tH3 | aH2 | Admixed | Yunnan, China | WSGR | YN |
|  |  | Miduxiangsu | H6 | tH4 | aH1 | Admixed | Yunnan, China | WSGR | YN |
|  |  | Wenshanhongli | H14 | tH5 | aH9 | Admixed | Yunnan, China | WSGR | YN |
|  |  | Lijiangbaili | H14 | tH5 | aH9 | Yellow | Yunnan, China | WSGR | YN |
|  |  | Lijiangzhima | H7 | tH4 | aH2 | Admixed | Yunnan, China | WSGR | YN |
|  |  | Qiubaili | H14 | tH5 | aH9 | Admixed | Yunnan, China | WSGR | YN |
|  |  | Bakesi | H3 | tH3 | aH2 | Admixed | Yunnan, China | CPGR | YN |
|  |  | Luliangtiansuan | H14 | tH5 | aH9 | Admixed | Yunnan, China | CPGR | YN |
|  |  | Changshuihuoba | H9 | tH4 | aH9 | Admixed | Yunnan, China | WSGR | YN |
|  |  | Chonghuadali | H3 | tH3 | aH2 | Purple | Sichuan, China | CPGR | SC |
|  |  | Damali | H14 | tH5 | aH9 | Admixed | Sichuan, China | CPGR | SC |
|  |  | Hongxiang | H4 | tH3 | aH9 | Admixed | Sichuan, China | CPGR | SC |
|  |  | Cangxixueli | H11 | tH5 | aH2 | Admixed | Sichuan, China | CPGR | SC |
|  |  | Duanbajitui | H14 | tH5 | aH9 | Admixed | Sichuan, China | CPGR | SC |
|  |  | Hongpisu | H14 | tH5 | aH9 | Yellow | Sichuan, China | CPGR | SC |
|  |  | Chenjiadama | H14 | tH5 | aH9 | Admixed | Sichuan, China | CPGR | SC |
|  |  | Damali | H14 | tH5 | aH9 | Admixed | Sichuan, China | CPGR | SC |
|  |  | Huangjitui | H4 | tH3 | aH9 | Admixed | Sichuan, China | CPGR | SC |
|  |  | Xiangma | H14 | tH5 | aH9 | Admixed | Sichuan, China | CPGR | SC |
|  |  | Xinxiang | H3 | tH3 | aH2 | Admixed | Sichuan, China | CPGR | SC |
|  |  | Yingxue | H14 | tH5 | aH9 | Admixed | Sichuan, China | CPGR | SC |
|  |  | Gongchuan | H6 | tH4 | aH1 | Admixed | Sichuan, China | WSGR | SC |
|  |  | Hanyuanbai | H14 | tH5 | aH9 | Admixed | Sichuan, China | WSGR | SC |
|  |  | Fangongli | H14 | tH5 | aH9 | Admixed | Sichuan, China | WSGR | SC |
|  |  | Xichanghoushanli | H14 | tH5 | aH9 | Admixed | Sichuan, China | WSGR | SC |
|  |  | Jinchuanmugua | H7 | tH4 | aH2 | Admixed | Sichuan, China | CPGR | SC |
|  |  | Jinchuanxueli | H14 | tH5 | aH9 | Admixed | Sichuan, China | CPGR | SC |
|  |  | Hongshao | H14 | tH5 | aH9 | Admixed | Sichuan, China | CPGR | SC |
|  |  | Piaoxinghuoba | H15 | tH5 | aH10 | Admixed | Sichuan, China | WSGR | SC |
|  |  | Hongma | H9 | tH4 | aH9 | Admixed | Sichuan, China | WSGR | SC |
|  |  | Xueshan1 | H14 | tH5 | aH9 | Admixed | Sichuan, China | WSGR | SC |
|  |  | Bayuexue | H6 | tH4 | aH1 | Admixed | Sichuan, China | WSGR | SC |
|  |  | Caimianya | H6 | tH4 | aH1 | Purple | Sichuan, China | WSGR | SC |
|  |  | Gongchuan | H14 | tH5 | aH9 | Purple | Sichuan, China | WSGR | SC |
|  |  | Jinhuali | H11 | tH5 | aH2 | Admixed | Sichuan, China | WSGR | SC |
|  |  | Hongpijidan | H3 | tH3 | aH2 | Admixed | Sichuan, China | WSGR | SC |
|  |  | Hongpisu | H11 | tH5 | aH2 | Admixed | Sichuan, China | WSGR | SC |
|  |  | Xianghua | H4 | tH3 | aH9 | Admixed | Sichuan, China | WSGR | SC |
|  |  | Yingtoubai | H6 | tH4 | aH1 | Admixed | Hubei, China | WSGR | HHJ |
|  |  | Guoguoli | H6 | tH4 | aH1 | Admixed | Hubei, China | WSGR | HHJ |
|  |  | Houzui | H16 | tH5 | aH36 | Admixed | Hubei, China | WSGR | HHJ |
|  |  | Shuima | H6 | tH4 | aH1 | Admixed | Hubei, China | WSGR | HHJ |
|  |  | Tiantanggengzi | H18 | tH20 | aH4 | Admixed | Hubei, China | WSGR | HHJ |
|  |  | Wuyuejin | H14 | tH5 | aH9 | Green | Hubei, China | WSGR | HHJ |
|  |  | Xianfengxueping | H14 | tH5 | aH9 | Admixed | Hubei, China | WSGR | HHJ |
|  |  | Zaoyangzhimasu | H14 | tH5 | aH9 | Purple | Hubei, China | WSGR | HHJ |
|  |  | Yuananwangshuibai | H14 | tH5 | aH9 | Purple | Hubei, China | WSGR | HHJ |
|  |  | Linwuxiangli | H18 | tH20 | aH4 | Purple | Hunan, China | WSGR | HHJ |
|  |  | Dabali | H6 | tH4 | aH1 | Purple | Hubei, China | WSGR | HHJ |
|  |  | Banjinli | H6 | tH4 | aH1 | Admixed | Hubei, China | WSGR | HHJ |
|  |  | Linwuxianghuali | H6 | tH4 | aH1 | Admixed | Hunan, China | WSGR | HHJ |
|  |  | Yizhangdaqingli | H14 | tH5 | aH9 | Admixed | Hunan, China | WSGR | HHJ |
|  |  | Yizhangduanbazao | H14 | tH5 | aH9 | Admixed | Hunan, China | WSGR | HHJ |
|  |  | Linwuzaomali | H14 | tH5 | aH9 | Admixed | Hunan, China | WSGR | HHJ |
|  |  | Linwushexiangli | H18 | tH20 | aH4 | Admixed | Hunan, China | WSGR | HHJ |
|  |  | Tianxiaoli | H16 | tH5 | aH36 | Admixed | Hunan, China | WSGR | HHJ |
|  |  | Jiangwanxiaohongli | H6 | tH4 | aH1 | Admixed | Jiangxi, China | CPGR | HHJ |
|  |  | Dayexueli | H14 | tH5 | aH9 | Admixed | Jiangxi, China | WSGR | HHJ |
|  |  | Qiubailu | H6 | tH4 | aH1 | Admixed | Jiangxi, China | WSGR | HHJ |
|  |  | Wuyuantangli | H7 | tH4 | aH2 | Admixed | Jiangxi, China | WSGR | HHJ |
|  |  | Cupisu | H6 | tH4 | aH1 | Admixed | Jiangxi, China | CPGR | HHJ |
|  |  | Kuixingmake | H6 | tH4 | aH1 | Purple | Jiangxi, China | WSGR | HHJ |
|  |  | Puli | H6 | tH4 | aH1 | Admixed | Jiangxi, China | WSGR | HHJ |
|  |  | Shangraocupixiwu | H6 | tH4 | aH1 | Admixed | Jiangxi, China | WSGR | HHJ |
|  |  | Shangraojiangwanxipili | H6 | tH4 | aH1 | Admixed | Jiangxi, China | WSGR | HHJ |
|  |  | Pulixiao | H6 | tH4 | aH1 | Admixed | Jiangxi, China | CPGR | HHJ |
|  |  | Cubingsu | H6 | tH4 | aH1 | Admixed | Jiangxi, China | WSGR | HHJ |
|  |  | Wuyuansuli | H6 | tH4 | aH1 | Admixed | Jiangxi, China | WSGR | HHJ |
|  |  | Puli | H6 | tH4 | aH1 | Admixed | Jiangxi, China | CPGR | HHJ |
|  |  | Zaoli | H7 | tH4 | aH2 | Admixed | Jiangxi, China | WSGR | HHJ |
|  |  | Hehua | H6 | tH4 | aH1 | Admixed | Jiangxi, China | WSGR | HHJ |
|  |  | Xipixijiangwu | H6 | tH4 | aH1 | Admixed | Jiangxi, China | CPGR | HHJ |
| *P. pyrifolia* | 125 | Qingpicao | H6 | tH4 | aH1 | Admixed | Anhui, China | CPGR | AJ |
| Chinese white pear | | Tiepi | H14 | tH5 | aH9 | Purple | Anhui, China | CPGR | AJ |
|  |  | Xijiangwu | H6 | tH4 | aH1 | Admixed | Anhui, China | CPGR | AJ |
|  |  | Zisu | H14 | tH5 | aH9 | Purple | Anhui, China | CPGR | AJ |
|  |  | Muguali | H3 | tH3 | aH2 | Admixed | Anhui, China | WSGR | AJ |
|  |  | Ehuang | H6 | tH4 | aH1 | Purple | Anhui, China | WSGR | AJ |
|  |  | Xiangmian | H6 | tH4 | aH1 | Admixed | Anhui, China | WSGR | AJ |
|  |  | Dangshanmatihuang | H6 | tH4 | aH1 | Purple | Anhui, China | WSGR | AJ |
|  |  | Lengsu | H10 | tH5 | aH1 | Admixed | Anhui, China | WSGR | AJ |
|  |  | Youpisu | H6 | tH4 | aH1 | Purple | Anhui, China | WSGR | AJ |
|  |  | Xiusu | H6 | tH4 | aH1 | Purple | Anhui, China | WSGR | AJ |
|  |  | Jizhuahuang | H14 | tH5 | aH9 | Admixed | Anhui, China | WSGR | AJ |
|  |  | Dangshansuli | H6 | tH4 | aH1 | Purple | Anhui, China | WSGR | AJ |
|  |  | Esu | H14 | tH5 | aH9 | Purple | Jiangsu, China | CPGR | AJ |
|  |  | Mingjiang | H14 | tH5 | aH9 | Purple | Jiangsu, China | CPGR | AJ |
|  |  | Siyangqingli | H6 | tH4 | aH1 | Admixed | Jiangsu, China | CPGR | AJ |
|  |  | Tianchengzi | H6 | tH4 | aH1 | Purple | Jiangsu, China | CPGR | AJ |
|  |  | Ruanzhiqing | H14 | tH5 | aH9 | Admixed | Jiangsu, China | CPGR | AJ |
|  |  | Lianyunganghuangli | H6 | tH4 | aH1 | Purple | Jiangsu, China | CPGR | AJ |
|  |  | Dashuihe | H6 | tH4 | aH1 | Admixed | Jiangsu, China | CPGR | AJ |
|  |  | Hongmacha | H6 | tH4 | aH1 | Admixed | Jiangsu, China | CPGR | AJ |
|  |  | Huangmacha | H6 | tH4 | aH1 | Admixed | Jiangsu, China | CPGR | AJ |
|  |  | Misu | H7 | tH4 | aH2 | Admixed | Jiangsu, China | CPGR | AJ |
|  |  | Dashuihe | H6 | tH4 | aH1 | Admixed | Jiangsu, China | CPGR | AJ |
|  |  | Haitangsu | H14 | tH5 | aH9 | Purple | Jiangsu, China | CPGR | AJ |
|  |  | Yingzhiqing | H6 | tH4 | aH1 | Admixed | Jiangsu, China | CPGR | AJ |
|  |  | Jingchuan | H3 | tH3 | aH2 | Admixed | Gansu, China | CPGR | GQ |
|  |  | Lintaoxueli | H13 | tH5 | aH8 | Admixed | Gansu, China | GPI | GQ |
|  |  | Guanghehongxiaoli | H3 | tH3 | aH2 | Admixed | Gansu, China | GPI | GQ |
|  |  | Kanglebingtangli | H6 | tH4 | aH1 | Green | Gansu, China | GPI | GQ |
|  |  | Lintaomuguali | H6 | tH4 | aH1 | Green | Gansu, China | GPI | GQ |
|  |  | Taojiazhuanghongxiaoli | H3 | tH3 | aH2 | Admixed | Gansu, China | GPI | GQ |
|  |  | Gangubapanli | H3 | tH3 | aH2 | Admixed | Gansu, China | GPI | GQ |
|  |  | Wudumaili | H3 | tH3 | aH2 | Admixed | Gansu, China | GPI | GQ |
|  |  | Ganguyuanli | H3 | tH3 | aH2 | Admixed | Gansu, China | GPI | GQ |
|  |  | Wushanyanguohong | H3 | tH3 | aH2 | Admixed | Gansu, China | GPI | GQ |
|  |  | Jingyuandadongguoli | H3 | tH3 | aH2 | Admixed | Gansu, China | GPI | GQ |
|  |  | Gangujinpingli | H3 | tH3 | aH2 | Admixed | Gansu, China | GPI | GQ |
|  |  | Longxidongjinping | H3 | tH3 | aH2 | Admixed | Gansu, China | GPI | GQ |
|  |  | Lixiandongguoli | H19 | tH21 | aH8 | Admixed | Gansu, China | GPI | GQ |
|  |  | Gangudongjinping | H3 | tH3 | aH2 | Admixed | Gansu, China | GPI | GQ |
|  |  | Wudutianli | H3 | tH3 | aH2 | Admixed | Gansu, China | GPI | GQ |
|  |  | Sanjiajimuguoli | H3 | tH3 | aH2 | Admixed | Gansu, China | GPI | GQ |
|  |  | Wudujituili | H3 | tH3 | aH2 | Admixed | Gansu, China | GPI | GQ |
|  |  | Longxihongjinping | H3 | tH3 | aH2 | Admixed | Gansu, China | GPI | GQ |
|  |  | Lixianxinbapanli | H7 | tH4 | aH2 | Admixed | Gansu, China | GPI | GQ |
|  |  | Ganguhongxia | H3 | tH3 | aH2 | Admixed | Gansu, China | GPI | GQ |
|  |  | Lixianlaobapanli | H3 | tH3 | aH2 | Admixed | Gansu, China | GPI | GQ |
|  |  | Longxisaminglali | H6 | tH4 | aH1 | Purple | Gansu, China | GPI | GQ |
|  |  | Kaichangli | H7 | tH4 | aH2 | Green | Gansu, China | GPI | GQ |
|  |  | Longxijituili | H3 | tH3 | aH2 | Admixed | Gansu, China | GPI | GQ |
|  |  | Miandanzili | H3 | tH3 | aH2 | Admixed | Gansu, China | GPI | GQ |
|  |  | Longxiwaibazili | H6 | tH4 | aH1 | Admixed | Gansu, China | GPI | GQ |
|  |  | Dongguoli | H3 | tH3 | aH2 | Admixed | Gansu, China | GPI | GQ |
|  |  | Bingtang | H3 | tH3 | aH2 | Admixed | Qinghai, China | GPI | GQ |
|  |  | Tieli | H14 | tH5 | aH9 | Purple | Shanxi, China | Shanxi, China | SS |
|  |  | Youli | H2 | tH3 | aH1 | Admixed | Shanxi, China | Shanxi, China | SS |
|  |  | Xiaowowo | H10 | tH5 | aH1 | Purple | Shanxi, China | Shanxi, China | SS |
|  |  | Dawowo | H6 | tH4 | aH1 | Green | Shanxi, China | Shanxi, China | SS |
|  |  | Gaodingbai | H10 | tH5 | aH1 | Admixed | Shanxi, China | Shanxi, China | SS |
|  |  | Tianli | H14 | tH5 | aH9 | Purple | Shanxi, China | Shanxi, China | SS |
|  |  | Gaopingdahuangli | H6 | tH4 | aH1 | Purple | Shanxi, China | Shanxi, China | SS |
|  |  | Jinchengxia | H3 | tH3 | aH2 | Admixed | Shanxi, China | Shanxi, China | SS |
|  |  | Pingli | H7 | tH4 | aH2 | Purple | Shanxi, China | Shanxi, China | SS |
|  |  | Suangelu | H6 | tH4 | aH1 | Purple | Shanxi, China | Shanxi, China | SS |
|  |  | Ganzi | H3 | tH3 | aH2 | Admixed | Shanxi, China | Shanxi, China | SS |
|  |  | Chuili | H3 | tH3 | aH2 | Admixed | Shanxi, China | Shanxi, China | SS |
|  |  | Benli | H14 | tH5 | aH9 | Admixed | Shanxi, China | Shanxi, China | SS |
|  |  | Tutouhong | H6 | tH4 | aH1 | Admixed | Shanxi, China | Shanxi, China | SS |
|  |  | Huachangba | H3 | tH3 | aH2 | Purple | Shanxi, China | Shanxi, China | SS |
|  |  | Xiaoli | H7 | tH4 | aH2 | Admixed | Shanxi, China | Shanxi, China | SS |
|  |  | Yuanpinghong | H3 | tH3 | aH2 | Purple | Shanxi, China | Shanxi, China | SS |
|  |  | Dali | H3 | tH3 | aH2 | Purple | Shanxi, China | Shanxi, China | SS |
|  |  | Youli | H3 | tH3 | aH2 | Admixed | Shanxi, China | Shanxi, China | SS |
|  |  | Binxianlaoyisheng | H14 | tH5 | aH9 | Admixed | Shaanxi, China | CPGR | SS |
|  |  | Wanrongjinli | H6 | tH4 | aH1 | Purple | Shanxi, China | Shanxi, China | SS |
|  |  | Fengxianjitui | H7 | tH4 | aH2 | Purple | Shaanxi, China | CPGR | SS |
|  |  | Xiangchun | H2 | tH3 | aH1 | Purple | Shaanxi, China | CPGR | SS |
|  |  | Xiaojin | H14 | tH5 | aH9 | Admixed | Shaanxi, China | CPGR | SS |
|  |  | Hongboli | H6 | tH4 | aH1 | Admixed | Shaanxi, China | CPGR | SS |
|  |  | Shuiyisheng | H14 | tH5 | aH9 | Purple | Shaanxi, China | CPGR | SS |
|  |  | Xiali | H6 | tH4 | aH1 | Purple | Shaanxi, China | CPGR | SS |
|  |  | Huangxianchangba | H6 | tH4 | aH1 | Admixed | Shandong, China | CPGR | SHH |
|  |  | Boshanchi | H14 | tH5 | aH9 | Purple | Shandong, China | CPGR | SHH |
|  |  | Jinanxiaobai | H6 | tH4 | aH1 | Purple | Shandong, China | CPGR | SHH |
|  |  | Cuozi | H14 | tH5 | aH9 | Purple | Shandong, China | CPGR | SHH |
|  |  | Jinanxiaohuang | H6 | tH4 | aH1 | Purple | Shandong, China | CPGR | SHH |
|  |  | Cili | H14 | tH5 | aH9 | Purple | Shandong, China | CPGR | SHH |
|  |  | Daaoao | H14 | tH5 | aH9 | Purple | Shandong, China | CPGR | SHH |
|  |  | Qixiaxiaoxiangshui | H14 | tH5 | aH9 | Purple | Shandong, China | CPGR | SHH |
|  |  | Zimuli | H14 | tH5 | aH9 | Admixed | Shandong, China | CPGR | SHH |
|  |  | Huangxianqiuli | H6 | tH4 | aH1 | Purple | Shandong, China | CPGR | SHH |
|  |  | Canglimatanggengzi | H12 | tH5 | aH4 | Purple | Hebei, China | CPGR | SHH |
|  |  | Fojianxi | H6 | tH4 | aH1 | Purple | Hebei, China | CPGR | SHH |
|  |  | Jidanguan | H14 | tH5 | aH9 | Admixed | Hebei, China | CPGR | SHH |
|  |  | Shuibai | H6 | tH4 | aH1 | Admixed | Hebei, China | CPGR | SHH |
|  |  | Taihuang | H6 | tH4 | aH1 | Admixed | Hebei, China | CPGR | SHH |
|  |  | Shuixiang | H10 | tH5 | aH1 | Purple | Hebei, China | CPGR | SHH |
|  |  | Yuanxiang | H3 | tH3 | aH2 | Admixed | Hebei, China | CPGR | SHH |
|  |  | Youping | H14 | tH5 | aH9 | Purple | Hebei, China | CPGR | SHH |
|  |  | Xinglongma | H6 | tH4 | aH1 | Admixed | Hebei, China | CPGR | SHH |
|  |  | Mili | H14 | tH5 | aH9 | Admixed | Hebei, China | CPGR | SHH |
|  |  | Pingdingcui | H6 | tH4 | aH1 | Purple | Hebei, China | CPGR | SHH |
|  |  | Yalao | H2 | tH3 | aH1 | Purple | Hebei, China | CPGR | SHH |
|  |  | Yangbaixiao | H14 | tH5 | aH9 | Purple | Hebei, China | CPGR | SHH |
|  |  | Yinbai | H3 | tH3 | aH2 | Admixed | Hebei, China | CPGR | SHH |
|  |  | Banjinsu | H3 | tH3 | aH2 | Admixed | Hebei, China | CPGR | SHH |
|  |  | Boli | H6 | tH4 | aH1 | Admixed | Hebei, China | CPGR | SHH |
|  |  | Fuli | H14 | tH5 | aH9 | Admixed | Hebei, China | CPGR | SHH |
|  |  | Mapiao | H6 | tH4 | aH1 | Green | Hebei, China | CPGR | SHH |
|  |  | Yali | H6 | tH4 | aH1 | Purple | Hebei, China | CPGR | SHH |
|  |  | Jinzhuguo | H2 | tH3 | aH1 | Admixed | Henan, China | CPGR | SHH |
|  |  | Xuehua | H6 | tH4 | aH1 | Purple | Hebei, China | CPGR | SHH |
|  |  | Banjinsu | H3 | tH3 | aH2 | Purple | Hebei, China | CPGR | SHH |
|  |  | Eli | H6 | tH4 | aH1 | Purple | Liaoning, China | CPGR | JL |
|  |  | Etou | H14 | tH5 | aH9 | Admixed | Liaoning, China | CPGR | JL |
|  |  | Fenhongxiao | H6 | tH4 | aH1 | Purple | Liaoning, China | CPGR | JL |
|  |  | Jinchuizi | H6 | tH4 | aH1 | Purple | Liaoning, China | CPGR | JL |
|  |  | Haichengcha | H6 | tH4 | aH1 | Purple | Liaoning, China | CPGR | JL |
|  |  | Xiuyanci | H6 | tH4 | aH1 | Green | Liaoning, China | CPGR | JL |
|  |  | Shuihongxiao | H6 | tH4 | aH1 | Admixed | Liaoning, China | CPGR | JL |
|  |  | Hongtang | H14 | tH5 | aH9 | Admixed | Liaoning, China | CPGR | JL |
|  |  | Xiehuatian | H6 | tH4 | aH1 | Admixed | Liaoning, China | CPGR | JL |
|  |  | Yanbianmingyue | H14 | tH5 | aH9 | Admixed | Jilin, China | CPGR | JL |
|  |  | Yanbiandahuang | H14 | tH5 | aH9 | Purple | Jilin, China | CPGR | JL |
| *P. ussuriensis* | 22 | Hongbalixiang | H14 | tH5 | aH9 | Red | Liaoning, China | CPGR | UP |
| Ussurian pears |  | Daxiangshui | H6 | tH4 | aH1 | Red | Liaoning, China | CPGR | UP |
|  |  | Huagai | H1 | tH1 | aH8 | Red | Liaoning, China | CPGR | UP |
|  |  | Jinxishanli | H1 | tH1 | aH8 | Admixed | Liaoning, China | Liaoning, China | UP |
|  |  | Reqiuzi | H14 | tH5 | aH9 | Red | Liaoning, China | CPGR | UP |
|  |  | Tianqiuzi | H14 | tH5 | aH9 | Admixed | Liaoning, China | CPGR | UP |
|  |  | Shanli | H14 | tH5 | aH9 | Admixed | Liaoning, China | CPGR | UP |
|  |  | Jianbali | H14 | tH5 | aH9 | Admixed | Liaoning, China | CPGR | UP |
|  |  | Xingchengxiehuatian | H14 | tH5 | aH9 | Admixed | Liaoning, China | CPGR | UP |
|  |  | Shanli | H6 | tH4 | aH1 | Admixed | Hebei, China | CPGR | UP |
|  |  | Jingbaili | H14 | tH5 | aH9 | Admixed | Beijing, China | CPGR | UP |
|  |  | Ruanerli | H16 | tH5 | aH36 | Admixed | Gansu, China | CPGR | UP |
|  |  | Qingmian | H3 | tH3 | aH2 | Admixed | Gansu, China | CPGR | UP |
|  |  | Manyuanxiang | H14 | tH5 | aH9 | Admixed | Liaoning, China | CPGR | UP |
|  |  | Nanguoli | H14 | tH5 | aH9 | Admixed | Liaoning, China | CPGR | UP |
|  |  | Longxiang | H6 | tH4 | aH1 | Admixed | Liaoning, China | CPGR | UP |
|  |  | Balixiang | H14 | tH5 | aH9 | Red | Liaoning, China | CPGR | UP |
|  |  | Yaguang | H14 | tH5 | aH9 | Red | Beijing, China | CPGR | UP |
|  |  | Saozhoumiaozi | H6 | tH4 | aH1 | Admixed | Hebei, China | CPGR | UP |
|  |  | Xiaoxiangshui | H1 | tH1 | aH8 | Admixed | Liaoning, China | Liaoning, China | UP |
|  |  | Yanbiandajianba | H6 | tH4 | aH1 | Red | Liaoning, China | Liaoning, China | UP |
|  |  | Fujiahuoba | H6 | tH4 | aH1 | Red | Liaoning, China | Liaoning, China | UP |
| *P. ussuriensis* | 18 | *P. ussuriensis*1 | H20 | tH1 | aH6 | Red | Heilongjiang, China | HAS-HAAS | WUP |
| Wild accessions |  | *P. ussuriensis*2 | H20 | tH1 | aH6 | Red | Jilin, China | HAS-HAAS | WUP |
|  |  | *P. ussuriensis*3 | H21 | tH1 | aH38 | Red | Heilongjiang, China | CSUFT | WUP |
|  |  | *P. ussuriensis*4 | H22 | tH1 | aH1 | Red | Jilin, China | CSUFT | WUP |
|  |  | *P. ussuriensis*5 | H23 | tH1 | aH37 | Red | Inner mongolia, China | CSUFT | WUP |
|  |  | *P. ussuriensis*6 | H20 | tH1 | aH6 | Red | Heilongjiang, China | HAS-HAAS | WUP |
|  |  | *P. ussuriensis*7 | H20 | tH1 | aH6 | Red | Jilin, China | HAS-HAAS | WUP |
|  |  | *P. ussuriensis*8 | H20 | tH1 | aH6 | Red | Jilin, China | HAS-HAAS | WUP |
|  |  | *P. ussuriensis*9 | H1 | tH1 | aH8 | Red | Jilin, China | HAS-HAAS | WUP |
|  |  | *P. ussuriensis*10 | H1 | tH1 | aH8 | Red | Heilongjiang, China | HAS-HAAS | WUP |
|  |  | *P. ussuriensis*11 | H3 | tH3 | aH2 | Red | Heilongjiang, China | HAS-HAAS | WUP |
|  |  | *P. ussuriensis*12 | H20 | tH1 | aH6 | Red | Heilongjiang, China | HAS-HAAS | WUP |
|  |  | *P. ussuriensis*13 | H20 | tH1 | aH6 | Red | Heilongjiang, China | HAS-HAAS | WUP |
|  |  | *P. ussuriensis*14 | H20 | tH1 | aH6 | Red | Heilongjiang, China | HAS-HAAS | WUP |
|  |  | *P. ussuriensis*15 | H20 | tH1 | aH6 | Red | Heilongjiang, China | HAS-HAAS | WUP |
|  |  | *P. ussuriensis*16 | H20 | tH1 | aH6 | Red | Heilongjiang, China | HAS-HAAS | WUP |
|  |  | *P. ussuriensis*17 | H20 | tH1 | aH6 | Red | Heilongjiang, China | HAS-HAAS | WUP |
|  |  | *P. ussuriensis*18 | H20 | tH1 | aH6 | Red | Liaoning, China | HAS-HAAS | WUP |

The haplotypes of each cpDNA fragment were identified and named after the previous phylogeny study in *Pyrus* (Zheng et al., 2014). GPI: Gansu Academy of Agricultural Sciences, Lanzhou, Gansu Province, China; WSGR: Wuhan Sand Pear Germplasm Repository, Wuhan, Hubei Province, China; CPGR: Pear Germplasm Repository, Xingcheng, Liaoning Province, China; TU: Tottori University, Tottori, Japan; HSA-HAAS: Horticultural Sub-Academy, Heilongjiang Academy of Agricultural Sciences, Harbin, Heilongjiang, China; CSUFT: Central South University of Forestry and Technology, Changsha, Hunan Province, China.

**Supplementary table S2 Genetic diversity statistics of East Asian pear accessions based on 25 microsatellite loci.**

| Locus | Linkage Groups | Size | *N* | *Na* | *Ne* | *I* | *H_O_* | *H_E_* |
| --- | --- | --- | --- | --- | --- | --- | --- | --- |
| NAUpy45d | LG1 | 117-167 | 26 | 11.125 | 5.817 | 1.978 | 0.789 | 0.818 |
| CTG1064726 | LG1 | 191-311 | 25 | 10.563 | 5.904 | 1.971 | 0.494 | 0.807 |
| BGT23b | LG2 | 170-244 | 32 | 9.563 | 4.599 | 1.757 | 0.673 | 0.769 |
| KU10 | LG2 | 219-289 | 31 | 8.125 | 3.870 | 1.535 | 0.642 | 0.702 |
| NAUpy02E | LG2 | 206-242 | 23 | 6.375 | 2.728 | 1.241 | 0.541 | 0.594 |
| MES138 | LG3 | 153-209 | 23 | 7.688 | 4.273 | 1.633 | 0.719 | 0.763 |
| NAUpy36E | LG4 | 109-201 | 43 | 13.313 | 5.314 | 1.980 | 0.529 | 0.775 |
| NAUpy08T | LG4 | 153-253 | 34 | 13.813 | 8.043 | 2.279 | 0.704 | 0.866 |
| NAUpy28i | LG5 | 136-184 | 25 | 10.688 | 6.015 | 1.935 | 0.606 | 0.790 |
| NAUpy28R | LG7 | 107-159 | 20 | 12.938 | 6.723 | 2.129 | 0.714 | 0.834 |
| NH026a | LG10 | 115-175 | 23 | 5.625 | 3.082 | 1.272 | 0.389 | 0.635 |
| CH01F02 | LG11 | 139-209 | 30 | 14.000 | 7.549 | 2.266 | 0.830 | 0.862 |
| 28f4 | LG12 | 92-120 | 16 | 8.688 | 4.822 | 1.752 | 0.666 | 0.777 |
| NAUpy34m | LG13 | 118-164 | 22 | 8.750 | 3.803 | 1.600 | 0.603 | 0.701 |
| NH019b | LG15 | 167-203 | 19 | 4.688 | 2.290 | 1.004 | 0.291 | 0.546 |
| 02b1 | LG15 | 244-272 | 14 | 9.688 | 5.158 | 1.843 | 0.717 | 0.790 |
| KA14 | LG16 | 170-212 | 21 | 6.938 | 1.695 | 0.860 | 0.241 | 0.367 |
| NAUpy25n | LG17 | 126-228 | 43 | 10.875 | 5.789 | 1.969 | 0.749 | 0.810 |
| MES2 | Unknown | 174-238 | 24 | 10.813 | 6.442 | 2.018 | 0.797 | 0.828 |
| MES122 | Unknown | 92-140 | 21 | 7.875 | 4.338 | 1.646 | 0.684 | 0.750 |
| MES108 | Unknown | 186-226 | 21 | 9.250 | 5.576 | 1.876 | 0.750 | 0.812 |
| MES7 | Unknown | 115-165 | 21 | 9.563 | 3.387 | 1.538 | 0.448 | 0.666 |
| TXY94 | Unknown | 170-208 | 20 | 9.938 | 4.867 | 1.785 | 0.433 | 0.759 |
| TXY121 | Unknown | 109-143 | 15 | 11.188 | 6.056 | 2.019 | 0.502 | 0.824 |
| TXY104 | Unknown | 248-272 | 13 | 7.625 | 4.145 | 1.608 | 0.562 | 0.749 |
| Mean |  |  | 24 | 9.588 | 4.891 | 1.740 | 0.603 | 0.744 |

Locus “BGT”, “KA”, “KU”, “NB”, “NH” and “NAUpy” were derived from pear genome (Yamamoto et al. 2002a; Yamamoto et al. 2002b; Song et al. 2014), CH01F02, 28f4, 02b1, “CTG”, and “CN” SSRs from apple genome (Guilford et al. 1997; Gianfranceschi et al. 1998; Han et al. 2011), “MES” SSRs from Malus express sequence tags (ESTs) (Yao et al. 2010) and “TXY” SSRs from pear bud transcriptome unigenes (Yue et al. 2014) . Linkage Group information were referred to Chen et al. (2014). *N*: Number of alleles; *Na*: Observed number of alleles; *Ne*: Effective number of alleles; *I*: Shannon's Information index; *H*_E_: Expected heterozygosity; *H*_O_: Observed heterozygosity.

**Supplemental table S3** Analyses of molecular variance (AMOVAs) based on nuclear microsatellite (nSSR) data and chloroplast DNA data for geographical populations of cultivated *P. pyrifolia* (cultivar groups JP, CSP and CWP) and *P. ussuriensis* accessions, including cultivated *P. ussuriensis* (UP) and wild *P. ussuriensis* (WUP).

| Source of variance | nSSRs | | cpDNA | |
| --- | --- | --- | --- | --- |
|  | df | Percentage of total variance (%) | df | Percentage of total variance (%) |
| Cultivated *P. pyrifolia* + *P. ussuriensis* |  |  |  |  |
| Among species | 1 | 5.19 | 1 | 19.18 |
| Among populations within species | 14 | 5.89 | 14 | 14.96 |
| Within populations | 866 | 88.91 | 426 | 65.87 |
| Cultivated *P. pyrifolia* + Cultivated *P. ussuriensis* |  |  |  |  |
| Among cultivar groups | 1 | 2.21 | 1 | 7.81 |
| Among populations within cultivar group | 13 | 5.82 | 13 | 13.29 |
| Within populations | 831 | 91.96 | 409 | 78.9 |
| Cultivated *P. pyrifolia* |  |  |  |  |
| Among cultivar groups | 2 | 2.49 | 2 | 3.64 |
| Among populations within cultivar group | 11 | 4.32 | 11 | 12.06 |
| Within populations | 788 | 93.18 | 387 | 84.31 |
| *P. ussuriensis* |  |  |  |  |
| Among populations | 1 | 10.31 | 1 | 57.39 |
| Within populations | 78 | 89.69 | 39 | 42.61 |

All levels of differentiation are significant.

**Supplementary table S4** Geographical characterization of the STRUCTURE’s subgroup at *K* = 4 based on nSSR data.

| Group | | Number of genotypes | Population  Code | Number of plants | % STRUCTURE subgroup | % Geographic population |
| --- | --- | --- | --- | --- | --- | --- |
| Total Sample | | 441 | JPa | 29 | 6.58% | 100.00% |
|  |  |  | JPb | 26 | 5.90% | 100.00% |
|  |  |  | ZJ | 27 | 6.12% | 100.00% |
|  |  |  | FJ | 34 | 7.71% | 100.00% |
|  |  |  | GG | 35 | 7.94% | 100.00% |
|  |  |  | GZ | 32 | 7.26% | 100.00% |
|  |  |  | YN | 30 | 6.80% | 100.00% |
|  |  |  | SC | 29 | 6.58% | 100.00% |
|  |  |  | HHJ | 34 | 7.71% | 100.00% |
|  |  |  | AJ | 26 | 5.90% | 100.00% |
|  |  |  | GQ | 29 | 6.58% | 100.00% |
|  |  |  | SS | 27 | 6.12% | 100.00% |
|  |  |  | SHH | 22 | 4.99% | 100.00% |
|  |  |  | JL | 11 | 2.49% | 100.00% |
|  |  |  | UP | 22 | 4.99% | 100.00% |
|  |  |  | WUP | 18 | 4.08% | 100.00% |
| *K*=4 | *K*-4.1_green | 56 | JPa | 27 | 48.21% | 93.10% |
|  |  |  | JPb | 18 | 32.14% | 69.23% |
|  |  |  | ZJ | 3 | 5.36% | 11.11% |
|  |  |  | FJ | 1 | 1.79% | 2.94% |
|  |  |  | GG | 0 | 0.00% | 0.00% |
|  |  |  | GZ | 0 | 0.00% | 0.00% |
|  |  |  | YN | 0 | 0.00% | 0.00% |
|  |  |  | SC | 0 | 0.00% | 0.00% |
|  |  |  | HHJ | 1 | 1.79% | 2.94% |
|  |  |  | AJ | 0 | 0.00% | 0.00% |
|  |  |  | GQ | 3 | 5.36% | 10.34% |
|  |  |  | SS | 1 | 1.79% | 3.70% |
|  |  |  | SHH | 1 | 1.79% | 4.55% |
|  |  |  | JL | 1 | 1.79% | 9.09% |
|  |  |  | UP | 0 | 0.00% | 0.00% |
|  |  |  | WUP | 0 | 0.00% | 0.00% |
|  | *K*-4.2_yellow | 50 | JPa | 0 | 0.00% | 0.00% |
|  |  |  | JPb | 0 | 0.00% | 0.00% |
|  |  |  | ZJ | 0 | 0.00% | 0.00% |
|  |  |  | FJ | 11 | 22.00% | 32.35% |
|  |  |  | GG | 7 | 14.00% | 20.00% |
|  |  |  | GZ | 22 | 44.00% | 68.75% |
|  |  |  | YN | 9 | 18.00% | 30.00% |
|  |  |  | SC | 1 | 2.00% | 3.45% |
|  |  |  | HHJ | 0 | 0.00% | 0.00% |
|  |  |  | AJ | 0 | 0.00% | 0.00% |
|  |  |  | GQ | 0 | 0.00% | 0.00% |
|  |  |  | SS | 0 | 0.00% | 0.00% |
|  |  |  | SHH | 0 | 0.00% | 0.00% |
|  |  |  | JL | 0 | 0.00% | 0.00% |
|  |  |  | UP | 0 | 0.00% | 0.00% |
|  |  |  | WUP | 0 | 0.00% | 0.00% |
|  | *K*-4.3_Purple | 63 | JPa | 1 | 1.59% | 3.45% |
|  |  |  | JPb | 0 | 0.00% | 0.00% |
|  |  |  | ZJ | 2 | 3.17% | 7.41% |
|  |  |  | FJ | 2 | 3.17% | 5.88% |
|  |  |  | GG | 0 | 0.00% | 0.00% |
|  |  |  | GZ | 0 | 0.00% | 0.00% |
|  |  |  | YN | 1 | 1.59% | 3.33% |
|  |  |  | SC | 2 | 3.17% | 6.90% |
|  |  |  | HHJ | 5 | 7.94% | 14.71% |
|  |  |  | AJ | 12 | 19.05% | 46.15% |
|  |  |  | GQ | 1 | 1.59% | 3.45% |
|  |  |  | SS | 14 | 22.22% | 51.85% |
|  |  |  | SHH | 18 | 28.57% | 81.82% |
|  |  |  | JL | 5 | 7.94% | 45.45% |
|  |  |  | UP | 0 | 0.00% | 0.00% |
|  |  |  | WUP | 0 | 0.00% | 0.00% |
|  | *K*-4.4_Red | 26 | JPa | 0 | 0.00% | 0.00% |
|  |  |  | JPb | 0 | 0.00% | 0.00% |
|  |  |  | ZJ | 0 | 0.00% | 0.00% |
|  |  |  | FJ | 0 | 0.00% | 0.00% |
|  |  |  | GG | 0 | 0.00% | 0.00% |
|  |  |  | GZ | 0 | 0.00% | 0.00% |
|  |  |  | YN | 0 | 0.00% | 0.00% |
|  |  |  | SC | 0 | 0.00% | 0.00% |
|  |  |  | HHJ | 0 | 0.00% | 0.00% |
|  |  |  | AJ | 0 | 0.00% | 0.00% |
|  |  |  | GQ | 0 | 0.00% | 0.00% |
|  |  |  | SS | 0 | 0.00% | 0.00% |
|  |  |  | SHH | 0 | 0.00% | 0.00% |
|  |  |  | JL | 0 | 0.00% | 0.00% |
|  |  |  | UP | 8 | 30.77% | 36.36% |
|  |  |  | WUP | 18 | 69.23% | 100.00% |
|  | Admixed genotypes | 246 | JPa | 1 | 0.41% | 3.45% |
|  |  |  | JPb | 8 | 3.25% | 30.77% |
|  |  |  | ZJ | 22 | 8.94% | 81.48% |
|  |  |  | FJ | 20 | 8.13% | 58.82% |
|  |  |  | GG | 29 | 11.79% | 82.86% |
|  |  |  | GZ | 10 | 4.07% | 31.25% |
|  |  |  | YN | 20 | 8.13% | 66.67% |
|  |  |  | SC | 26 | 10.57% | 89.66% |
|  |  |  | HHJ | 27 | 10.98% | 79.41% |
|  |  |  | AJ | 13 | 5.28% | 50.00% |
|  |  |  | GQ | 26 | 10.57% | 89.66% |
|  |  |  | SS | 12 | 4.88% | 44.44% |
|  |  |  | SHH | 13 | 5.28% | 59.09% |
|  |  |  | JL | 5 | 2.03% | 45.45% |
|  |  |  | UP | 14 | 5.69% | 63.64% |
|  |  |  | WUP | 0 | 0.00% | 0.00% |

Groups *K*-4.1, *K*-4.2, *K*-4.3 and *K*-4.4 represent genetic groups were revealed by STRUCTURE (Pritchard et al., 2000) at *K*=4. Population codes were identified in Table S1.

**Supplementary table S5** Geographical characterization of the Bayesian’s clusters at *N* = 8 based on cpDNA data.

| Cluster | | Number of genotypes | Population Code | Number of plants | % Bayesian clusters | % Geographic population |
| --- | --- | --- | --- | --- | --- | --- |
|  |  |  |  |  |  |  |
| *N*=8 | *N*-8.1_Pink | 128 | JPa | 5 | 3.91% | 17.24% |
|  |  |  | JPb | 3 | 2.34% | 11.54% |
|  |  |  | ZJ | 5 | 3.91% | 17.86% |
|  |  |  | FJ | 4 | 3.13% | 11.43% |
|  |  |  | GG | 8 | 6.25% | 23.53% |
|  |  |  | GZ | 21 | 16.41% | 65.63% |
|  |  |  | YN | 15 | 11.72% | 50.00% |
|  |  |  | SC | 13 | 10.16% | 46.43% |
|  |  |  | HHJ | 8 | 6.25% | 23.53% |
|  |  |  | AJ | 7 | 5.47% | 26.92% |
|  |  |  | GQ | 4 | 3.13% | 13.79% |
|  |  |  | SS | 8 | 6.25% | 28.57% |
|  |  |  | SHH | 10 | 7.81% | 32.26% |
|  |  |  | JL | 4 | 3.13% | 36.36% |
|  |  |  | UP | 13 | 10.16% | 59.09% |
|  |  |  | WUP | 0 | 0.00% | 0.00% |
|  | *N-*8.2_Purple | 133 | JPa | 15 | 11.28% | 51.72% |
|  |  |  | JPb | 12 | 9.02% | 46.15% |
|  |  |  | ZJ | 15 | 11.28% | 53.57% |
|  |  |  | FJ | 11 | 8.27% | 31.43% |
|  |  |  | GG | 5 | 3.76% | 14.71% |
|  |  |  | GZ | 0 | 0.00% | 0.00% |
|  |  |  | YN | 2 | 1.50% | 6.67% |
|  |  |  | SC | 3 | 2.26% | 10.71% |
|  |  |  | HHJ | 21 | 15.79% | 61.76% |
|  |  |  | AJ | 17 | 12.78% | 65.38% |
|  |  |  | GQ | 2 | 1.50% | 6.90% |
|  |  |  | SS | 7 | 5.26% | 25.00% |
|  |  |  | SHH | 13 | 9.77% | 41.94% |
|  |  |  | JL | 6 | 4.51% | 54.55% |
|  |  |  | UP | 4 | 3.01% | 18.18% |
|  |  |  | WUP | 0 | 0.00% | 0.00% |
|  | *N*-8.3_Green | 62 | JPa | 9 | 14.52% | 31.03% |
|  |  |  | JPb | 8 | 12.90% | 30.77% |
|  |  |  | ZJ | 0 | 0.00% | 0.00% |
|  |  |  | FJ | 0 | 0.00% | 0.00% |
|  |  |  | GG | 0 | 0.00% | 0.00% |
|  |  |  | GZ | 5 | 8.06% | 15.63% |
|  |  |  | YN | 4 | 6.45% | 13.33% |
|  |  |  | SC | 0 | 0.00% | 0.00% |
|  |  |  | HHJ | 0 | 0.00% | 0.00% |
|  |  |  | AJ | 1 | 1.61% | 3.85% |
|  |  |  | GQ | 20 | 32.26% | 68.97% |
|  |  |  | SS | 7 | 11.29% | 25.00% |
|  |  |  | SHH | 5 | 8.06% | 16.13% |
|  |  |  | JL | 1 | 1.61% | 9.09% |
|  |  |  | UP | 2 | 3.23% | 9.09% |
|  |  |  | WUP | 0 | 0.00% | 0.00% |
|  | *N*-8.4_ Yellow | 52 | JPa | 0 | 0.00% | 0.00% |
|  |  |  | JPb | 2 | 3.85% | 7.69% |
|  |  |  | ZJ | 5 | 9.62% | 17.86% |
|  |  |  | FJ | 12 | 23.08% | 34.29% |
|  |  |  | GG | 16 | 30.77% | 47.06% |
|  |  |  | GZ | 4 | 7.69% | 12.50% |
|  |  |  | YN | 4 | 7.69% | 13.33% |
|  |  |  | SC | 3 | 5.77% | 10.71% |
|  |  |  | HHJ | 1 | 1.92% | 2.94% |
|  |  |  | AJ | 0 | 0.00% | 0.00% |
|  |  |  | GQ | 1 | 1.92% | 3.45% |
|  |  |  | SS | 3 | 5.77% | 10.71% |
|  |  |  | SHH | 1 | 1.92% | 3.23% |
|  |  |  | JL | 0 | 0.00% | 0.00% |
|  |  |  | UP | 0 | 0.00% | 0.00% |
|  |  |  | WUP | 0 | 0.00% | 0.00% |
|  | *N*-8.5_Brown | 15 | JPa | 0 | 0.00% | 0.00% |
|  |  |  | JPb | 0 | 0.00% | 0.00% |
|  |  |  | ZJ | 0 | 0.00% | 0.00% |
|  |  |  | FJ | 1 | 6.67% | 2.86% |
|  |  |  | GG | 4 | 26.67% | 11.76% |
|  |  |  | GZ | 0 | 0.00% | 0.00% |
|  |  |  | YN | 0 | 0.00% | 0.00% |
|  |  |  | SC | 0 | 0.00% | 0.00% |
|  |  |  | HHJ | 4 | 26.67% | 11.76% |
|  |  |  | AJ | 0 | 0.00% | 0.00% |
|  |  |  | GQ | 1 | 6.67% | 3.45% |
|  |  |  | SS | 0 | 0.00% | 0.00% |
|  |  |  | SHH | 2 | 13.33% | 6.45% |
|  |  |  | JL | 0 | 0.00% | 0.00% |
|  |  |  | UP | 2 | 13.33% | 9.09% |
|  |  |  | WUP | 1 | 6.67% | 5.56% |
|  | *N*-8.6_ Dark-blue | 16 | JPa | 0 | 0.00% | 0.00% |
|  |  |  | JPb | 0 | 0.00% | 0.00% |
|  |  |  | ZJ | 2 | 12.50% | 7.14% |
|  |  |  | FJ | 4 | 25.00% | 11.43% |
|  |  |  | GG | 1 | 6.25% | 2.94% |
|  |  |  | GZ | 1 | 6.25% | 3.13% |
|  |  |  | YN | 0 | 0.00% | 0.00% |
|  |  |  | SC | 4 | 25.00% | 14.29% |
|  |  |  | HHJ | 0 | 0.00% | 0.00% |
|  |  |  | AJ | 1 | 6.25% | 3.85% |
|  |  |  | GQ | 0 | 0.00% | 0.00% |
|  |  |  | SS | 2 | 12.50% | 7.14% |
|  |  |  | SHH | 0 | 0.00% | 0.00% |
|  |  |  | JL | 0 | 0.00% | 0.00% |
|  |  |  | UP | 0 | 0.00% | 0.00% |
|  |  |  | WUP | 1 | 6.25% | 5.56% |
|  | *N*-8.7_Light-green | 16 | JPa | 0 | 0.00% | 0.00% |
|  |  |  | JPb | 1 | 6.25% | 3.85% |
|  |  |  | ZJ | 1 | 6.25% | 3.57% |
|  |  |  | FJ | 3 | 18.75% | 8.57% |
|  |  |  | GG | 0 | 0.00% | 0.00% |
|  |  |  | GZ | 1 | 6.25% | 3.13% |
|  |  |  | YN | 5 | 31.25% | 16.67% |
|  |  |  | SC | 5 | 31.25% | 17.86% |
|  |  |  | HHJ | 0 | 0.00% | 0.00% |
|  |  |  | AJ | 0 | 0.00% | 0.00% |
|  |  |  | GQ | 0 | 0.00% | 0.00% |
|  |  |  | SS | 0 | 0.00% | 0.00% |
|  |  |  | SHH | 0 | 0.00% | 0.00% |
|  |  |  | JL | 0 | 0.00% | 0.00% |
|  |  |  | UP | 0 | 0.00% | 0.00% |
|  |  |  | WUP | 0 | 0.00% | 0.00% |
|  | *N*-8.8_Red | 19 | JPa | 0 | 0.00% | 0.00% |
|  |  |  | JPb | 0 | 0.00% | 0.00% |
|  |  |  | ZJ | 0 | 0.00% | 0.00% |
|  |  |  | FJ | 0 | 0.00% | 0.00% |
|  |  |  | GG | 0 | 0.00% | 0.00% |
|  |  |  | GZ | 0 | 0.00% | 0.00% |
|  |  |  | YN | 0 | 0.00% | 0.00% |
|  |  |  | SC | 0 | 0.00% | 0.00% |
|  |  |  | HHJ | 0 | 0.00% | 0.00% |
|  |  |  | AJ | 0 | 0.00% | 0.00% |
|  |  |  | GQ | 1 | 5.26% | 3.45% |
|  |  |  | SS | 1 | 5.26% | 3.57% |
|  |  |  | SHH | 0 | 0.00% | 0.00% |
|  |  |  | JL | 0 | 0.00% | 0.00% |
|  |  |  | UP | 1 | 5.26% | 4.55% |
|  |  |  | WUP | 16 | 84.21% | 88.89% |

Clusters *N*-8.1, *N*-8.2, *N*-8.3, *N*-8.4, *N*-8.5, *N*-8.6, *N*-8.7 and *N*-8.8 represent genetic clusters were revealed by BAPS6.0 (Corander et al., 2007) at *N*=8. Population codes were identified in Table S1.

**Supplementary table S6 Sequence polymorphisms of haplotypes detected in Chloroplast’ *trn*L-*trn*F and *acc*D-*psa*I from East Asian cultivated pears and wild *P. ussuriensis.***

| Origins | Haplotypes | | Polymorphism sites^†^ | | | | | | | | | | | | | | | | | |
| --- | --- | --- | --- | --- | --- | --- | --- | --- | --- | --- | --- | --- | --- | --- | --- | --- | --- | --- | --- | --- |
|  |  |  | *trn*L-*trn*F | | | | | | | *acc*D-*psa*I | | | | | | | | | | |
|  |  |  | C/T | A/T | INDEL^‡^ | T/G | INDEL^‡^ | A/C | G/A | INDEL^‡^ | INDEL^‡^ | A/C | INDEL^‡^ | A/G | C/T | INDEL^‡^ | INDEL^‡^ | INDEL^‡^ | T/C | C/G |
| Cultivated pears | H1 | tH1aH8 | C | A | 0 | T | 1^b^ | A | G | 0 | 1^d^ | A | 1^e^ | A | C | 0 | 0 | 0 | T | C |
| Cultivated pears | H2 | tH3aH1 | C | A | 1^a^ | T | 0 | C | A | 1^c^ | 1^d^ | A | 0 | A | C | 0 | 0 | 0 | T | C |
| Cultivated pears | H3 | tH3aH2 | C | A | 1^a^ | T | 0 | C | A | 0 | 1^d^ | A | 0 | A | C | 0 | 0 | 0 | T | C |
| Cultivated pears | H4 | tH3aH9 | C | A | 1^a^ | T | 0 | C | A | 0 | 1^d^ | A | 1^e^ | A | C | 0 | 1^g^ | 0 | T | G |
| Cultivated pears | H5 | tH5aH12 | C | A | 1^a^ | T | 0 | A | G | 0 | 1^d^ | A | 1^e^ | A | T | 0 | 1^g^ | 0 | C | C |
| Cultivated pears | H6 | tH4aH1 | C | A | 1^a^ | T | 0 | A | A | 1^c^ | 1^d^ | A | 0 | A | C | 0 | 0 | 0 | T | C |
| Cultivated pears | H7 | tH4aH2 | C | A | 1^a^ | T | 0 | A | A | 0 | 1^d^ | A | 0 | A | C | 0 | 0 | 0 | T | C |
| Cultivated pears | H8 | tH4aH4 | C | A | 1^a^ | T | 0 | A | A | 0 | 1^d^ | A | 1^e^ | G | C | 0 | 0 | 0 | T | C |
| Cultivated pears | H9 | tH4aH9 | C | A | 1^a^ | T | 0 | A | A | 0 | 1^d^ | A | 1^e^ | A | C | 0 | 1^g^ | 0 | T | G |
| Cultivated pears | H10 | tH5aH1 | C | A | 1^a^ | T | 0 | A | G | 1^c^ | 1^d^ | A | 0 | A | C | 0 | 0 | 0 | T | C |
| Cultivated pears | H11 | tH5aH2 | C | A | 1^a^ | T | 0 | A | G | 0 | 1^d^ | A | 0 | A | C | 0 | 0 | 0 | T | C |
| Cultivated pears | H12 | tH5aH4 | C | A | 1^a^ | T | 0 | A | G | 0 | 1^d^ | A | 1^e^ | G | C | 0 | 0 | 0 | T | C |
| Cultivated pears | H13 | tH5aH8 | C | A | 1^a^ | T | 0 | A | G | 0 | 1^d^ | A | 1^e^ | A | C | 0 | 0 | 0 | T | C |
| Cultivated pears | H14 | tH5aH9 | C | A | 1^a^ | T | 0 | A | G | 0 | 1^d^ | A | 1^e^ | A | C | 0 | 1^g^ | 0 | T | G |
| Cultivated pears | H15 | tH5aH10 | C | A | 1^a^ | T | 0 | A | G | 0 | 1^d^ | A | 1^e^ | A | C | 0 | 1^g^ | 0 | T | C |
| Cultivated pears | H16 | tH5aH36 | C | A | 1^a^ | T | 0 | A | G | 0 | 1^d^ | A | 1^e^ | A | C | 1^f^ | 0 | 0 | T | C |
| Cultivated pears | H17 | tH7aH10 | C | A | 1^a^ | G | 0 | A | G | 0 | 1^d^ | A | 1^e^ | A | C | 0 | 1^g^ | 0 | T | C |
| Cultivated pears | H18 | tH20aH4 | T | A | 1^a^ | T | 0 | A | G | 0 | 1^d^ | A | 1^e^ | G | C | 0 | 0 | 0 | T | C |
| Cultivated pears | H19 | tH21aH8 | C | T | 0 | T | 1^b^ | A | G | 0 | 1^d^ | A | 1^e^ | A | C | 0 | 0 | 0 | T | C |
| Wild *P. ussuriensis* | H20 | tH1aH6 | C | A | 0 | T | 1^b^ | A | G | 0 | 1^d^ | C | 1^e^ | A | C | 0 | 0 | 0 | T | C |
| Wild *P. ussuriensis* | H21 | tH1aH38 | C | A | 0 | T | 1^b^ | A | G | 0 | 1^d^ | A | 1^e^ | A | C | 0 | 0 | 1^h^ | T | C |
| Wild *P. ussuriensis* | H22 | tH1aH1 | C | A | 0 | T | 1^b^ | A | G | 1^c^ | 1^d^ | A | 0 | A | C | 0 | 0 | 0 | T | C |
| Wild *P. ussuriensis* | H23 | tH1aH37 | C | A | 0 | T | 1^b^ | A | G | 0 | 0 | C | 1^e^ | A | C | 0 | 0 | 0 | T | C |

All the polymorphism sites were got from sequences compared to tH5 or aH8, and ‘1/0’in the sequence indicated absence / presence of length mutation. a: 10bp deletion - TGGATCTGAG; b: 8bp insertion- TAATTGAC; c: T inserts; d: A deletion; e: 229bp deletions; f:23bp - ATGTAGAAAGATGAATTAGAAAC; g:22bp - TATTTATTGTATTTTATTAATT; h: 26bp - TTAATTAATATATATTTCTTAAATTA.

**Table S7** Statistical summary of chloroplast DNA haplotypes in East Asian pears

| Group  Code | Population  Code | Haplotype | | | | | | | | | | | | | | | | | | | | | | |
| --- | --- | --- | --- | --- | --- | --- | --- | --- | --- | --- | --- | --- | --- | --- | --- | --- | --- | --- | --- | --- | --- | --- | --- | --- |
|  |  | H1 | H2 | H3 | H4 | H5 | H6 | H7 | H8 | H9 | H10 | H11 | H12 | H13 | H14 | H15 | H16 | H17 | H18 | H19 | H20 | H21 | H22 | H23 |
| JP | JPa |  | 1 | 10 |  |  | 14 |  |  | 1 |  |  |  |  | 3 |  |  |  |  |  |  |  |  |  |
|  | JPb |  |  | 8 |  |  | 13 |  |  |  |  |  |  |  | 5 |  |  |  |  |  |  |  |  |  |
| CSP | ZJ |  |  | 5 |  |  | 14 |  |  | 1 | 3 |  |  |  | 4 |  |  |  |  |  |  |  |  |  |
|  | FJ |  |  |  |  |  | 11 | 12 |  |  |  | 4 |  | 1 | 3 | 3 |  |  |  |  |  |  |  |  |
|  | GG |  |  |  |  |  | 6 | 17 | 1 |  | 1 |  | 2 | 1 | 7 |  |  |  |  |  |  |  |  |  |
|  | GZ |  |  | 4 |  |  | 1 | 2 |  |  |  | 1 |  |  | 23 | 1 |  |  |  |  |  |  |  |  |
|  | YN |  |  | 4 |  | 1 | 2 | 2 |  | 3 |  |  |  |  | 16 | 1 |  | 1 |  |  |  |  |  |  |
|  | SC |  |  | 3 | 3 |  | 3 | 1 |  | 1 |  | 3 |  |  | 14 | 1 |  |  |  |  |  |  |  |  |
|  | HHJ |  |  |  |  |  | 19 | 2 |  |  |  |  |  |  | 8 |  | 2 |  | 3 |  |  |  |  |  |
| CWP | AJ |  |  | 1 |  |  | 16 | 1 |  |  | 1 |  |  |  | 7 |  |  |  |  |  |  |  |  |  |
|  | GQ |  |  | 21 |  |  | 4 | 2 |  |  |  |  |  | 1 |  |  |  |  |  | 1 |  |  |  |  |
|  | SS |  | 2 | 7 |  |  | 7 | 3 |  |  | 2 |  |  |  | 6 |  |  |  |  |  |  |  |  |  |
|  | SHH |  | 2 | 4 |  |  | 13 |  |  |  | 1 |  | 1 |  | 11 |  |  |  |  |  |  |  |  |  |
|  | JL |  |  |  |  |  | 7 |  |  |  |  |  |  |  | 4 |  |  |  |  |  |  |  |  |  |
| UP | UP | 3 |  | 1 |  |  | 6 |  |  |  |  |  |  |  | 11 |  | 1 |  |  |  |  |  |  |  |
| WUP | WUP | 2 |  | 1 |  |  |  |  |  |  |  |  |  |  |  |  |  |  |  |  | 12 | 1 | 1 | 1 |
| Total |  | 5 | 5 | 69 | 3 | 1 | 134 | 42 | 1 | 6 | 8 | 8 | 3 | 3 | 122 | 6 | 3 | 1 | 3 | 1 | 12 | 1 | 1 | 1 |

H1-H23 represent 23 haplotypes detected from combined chloroplast DNA sequences of *trn*L-*trn*F and *acc*D-*psa*I respectively.

**Supplementary table S8 Proportions of dominant haplotypes in East Asian pear cultivar groups.**

| Group  Code | *No.* | Haplotypes | | | |  |
| --- | --- | --- | --- | --- | --- | --- |
|  |  | H1 | H3 | H6 | H7 | H14 |
| JP | 55 |  | 18 (32.72%) | 27 (49.09%) |  | 8 (14.55%) |
| CSP | 221 |  | 16 (7.23%) | 56 (25.34%) | 36 (16.29%) | 75 (33.94%) |
| CWP | 125 |  | 33 (26.4%) | 47 (37.6%) | 6 (4.8%) | 28 (22.4%) |
| UP | 22 | 3 (13.64%) | 1 (4.55%) | 6 (27.18%) |  | 11 (50%) |
